# Seasonal, Oceanographic and Atmospheric Drivers of Diving Behaviour in a Temperate Seal Species Living in the High Arctic

**DOI:** 10.1371/journal.pone.0132686

**Published:** 2015-07-21

**Authors:** Marie-Anne Blanchet, Christian Lydersen, Rolf A. Ims, Kit M. Kovacs

**Affiliations:** 1 Norwegian Polar Institute, Fram Center, 9296 Tromsø, Norway; 2 Department of Arctic and Marine Biology, UiT-Arctic University of Norway, 9037 Tromsø, Norway; University of Waikato (National Institute of Water and Atmospheric Research), NEW ZEALAND

## Abstract

The harbour seal *(Phoca vitulina)* population in Svalbard marks the northernmost limit of the species’ range. This small population experiences environmental extremes in sea and air temperatures, sea ice cover and also in light regime for this normally temperate species. This study deployed Conductivity Temperature Depth Satellite Relay Data Loggers (CTD-SRDLs) on 30 adult and juvenile harbour seals in 2009 and 2010 to study their foraging behaviour across multiple seasons. A total of 189,104 dives and 16,640 CTD casts (mean depth 72 m ± 59) were recorded. Individuals dove to a mean depth of 41 m ± 24 with a maximum dive depth range of 24 – 403 m. Dives lasted on average 204 sec ± 120 with maximum durations ranging between 240 – 2,220 sec. Average daily depth and duration of dives, number of dives, time spent diving and dive time/surface time were influenced by date, while sex, age, sea-ice concentration and their interactions were not particularly influential. Dives were deeper (~150 m), longer (~480 sec), less numerous (~250 dives/day) and more pelagic during the winter/early spring compared to the fall and animals spent proportionally less time at the bottom of their dives during the winter. Influxes of warm saline water, corresponding to Atlantic Water characteristics, were observed intermittently at depths ~100 m during both winters in this study. The seasonal changes in diving behaviour were linked to average weekly wind stresses from the north or north-east, which induced upwelling events onto the shelf through offshore Ekman transport. During these events the shelf became flooded with AW from the West Spitsbergen Current, which presumably brought Atlantic fish species close to shore and within the seals’ foraging depth-range. Predicted increased in the influx of AW in this region are likely going to favour the growth and geographic expansion of this harbour seal population in the future.

## Introduction

Key foraging locations of top predators, such as sea birds and mammals, in marine environments have been shown to be tightly linked to oceanographic features and processes at various temporal and spatial scales [1–4]. Depending on their foraging strategy, marine mammals are influenced by either static or dynamic oceanographic features, or both. Animals foraging pelagically rely on predictable, albeit dynamic, features such as frontal structures [5], eddies, filaments [1,6,7], upwelling events or the stratification of the water column [8]; all of which can vary in time and space. On the other hand, benthic foragers tend to focus on particular topographical features of the sea bed such as sea mounts, trenches or shelf breaks that are known to concentrate nutrients [9–11]. Features such as these are often described as “biological hot-spots” because they serve to aggregate invertebrate and fish prey that in turn concentrate large predators [12]. Linkages between hydrographic features and biological production can however be challenging to demonstrate because of time lags existing between productivity and trophic dynamics the complexity of the relationships involved and the quality and the geographical scale of the available environmental data [1–4]. In addition, fine-scale oceanographic processes are rarely well described in remote Polar Regions where data, especially from during the winter in ice-filled waters, are limited or completely lacking [13]. However, recent advances in novel technologies allow animals to be fitted with multi-sensors instruments that collect data both on their own behaviour and the environmental conditions the animals are experiencing [14–16] at the same spatial and temporal scales.

**Fig 1:**
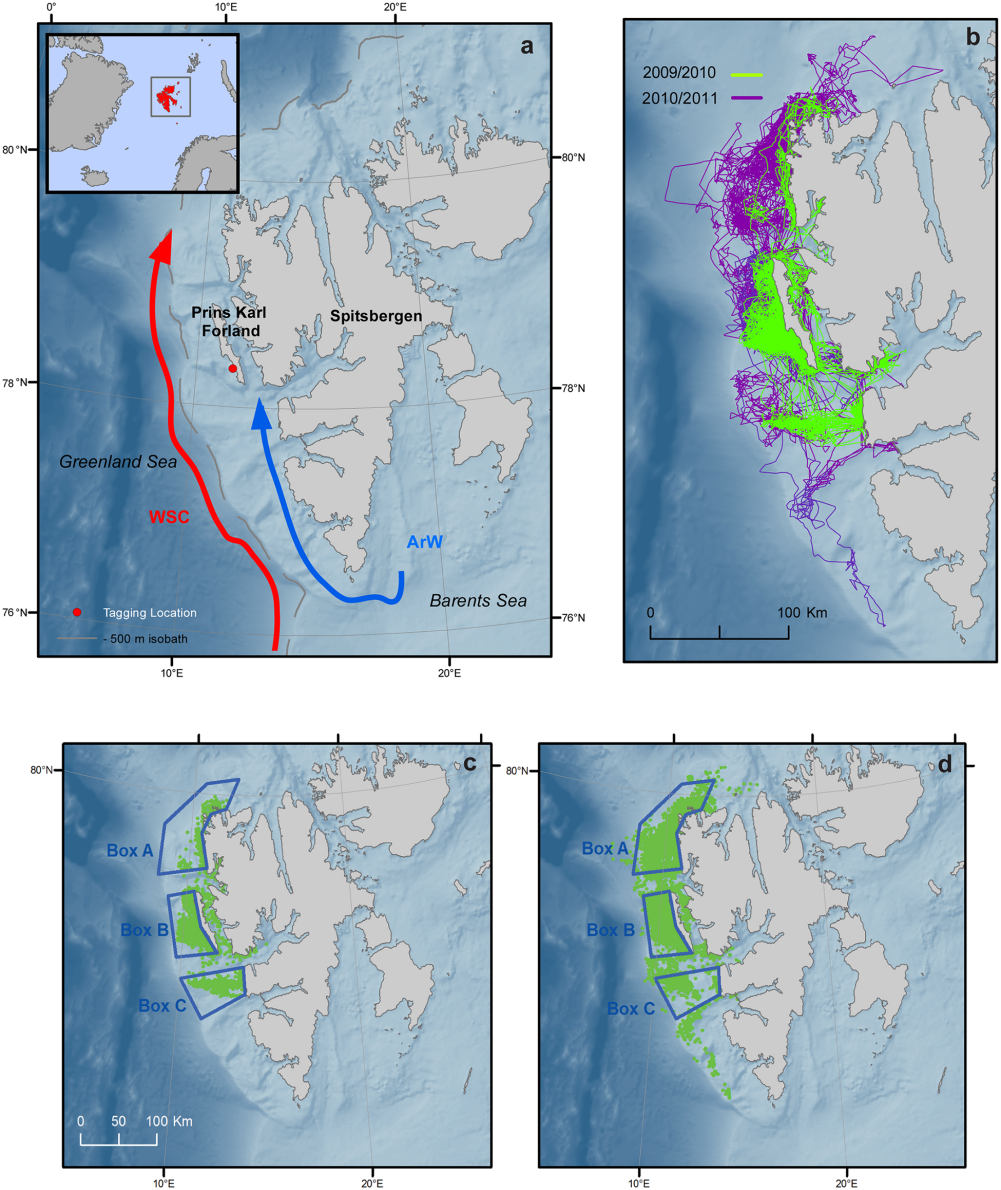
Map of the Svalbard Archipelago, Norway. (a) shows a map of the Svalbard Archipelago, the red dot represents the capture site on Forlandøyane west of Prins Karl Forland. The broken grey line represents the 500 m isobath delimiting the West Spistbergen Shelf. The West Spitsbergen Current (WSC) is represented by the red arrow flowing northwards along the continental slope; the coastal current is illustrated in blue. It is mainly composed of Arctic Water (ArW), which flows northwards along the west coast of Spitsbergen (modified from Nilsen et al. 2008). The Polar Front (not represented on the figure) is located at the shelf edge between the WSC and the coastal current (b) shows the filtered tracks of the 30 adult and juvenile harbour seals equipped with Conductivity Temperature Depth Satellite Relay Data Loggers (CTD-SRDLs) on Svalbard, Norway during 2009/2010 (green) and 2010/2011 (purple) overlaid on bathymetry (darker shades of blue indicate deeper water). **The inset** in (a) shows the Svalbard Archipelago’s geographical position. **The bottom panels** show the locations of Conductivity-Temperature-Depth (CTD) casts collected by the harbour seals during (c) 2009/2010 (d) and 2010/2011. Three selected geographical regions (A, B and C) used in oceanographic analyses are represented on these maps (c,d).

Harbour seals (*Phoca vitulina*) have one of the broadest distributions among the pinnipeds ranging from temperate areas to arctic waters of the North Pacific and the North Atlantic [17,18]. A few harbour seal populations inhabit arctic areas in the latter region, including southern Greenland [19]; northern Norway [20–22], Iceland [23] and the Murman area in north-western Russia [24,25]. Harbour seals are a coastal species [26] that is usually found within 50 km of their terrestrial haul-out sites [27,28]. The world’s northernmost population of harbour seal is located in Svalbard, where the seals reside year-round in the High Arctic [29–31]. The core of this population’s distribution is located on the west side of Prins Karls Forland (PKF) close to the shelf break west of Spitsbergen at about 78.5°N (Fig 1a). Individuals in this population experience extreme seasonal variation in the light regime, cold air and water temperatures, especially in winter, as well as considerable amounts of drifting sea ice [32]. Recent studies of the haul-out behaviour [33] and movement patterns [34] of this population have shown that these environmental parameters (light, sea-ice concentration and air pressure) have great influence on these facets of the behaviour of these seals. But, little is known about how environmental factors specifically influence their diving and foraging behaviour.

Environmental parameters, particularly water mass characteristics, influence the composition of potential prey communities for marine mammals. In other regions harbour seals are known to feed opportunistically on a wide variety of benthic and pelagic prey species and they show strong regional patterns [35,36]. Seasonal variation in harbour seal diet has also been documented repeatedly [35,37–41]; some of this seasonal variation has been linked to fish migratory patterns. Relatively little information is available regarding the diet or diving behaviour of harbour seals in Svalbard, and most of what is available is from the summer and early fall [29,30,42–44]; the winter and spring periods are especially poorly documented.

The Svalbard region, and in particular, the core of the area occupied by the local harbour seal population, is characterized by complex oceanographic conditions. Warm, saline, nutrient-rich Atlantic Water (AW) penetrates into the Arctic Ocean via the West Spitsbergen Current (WSC) along the shelf west of Spitsbergen (Fig 1a) and is one of the key factors that drives the high primary productivity in this region [45,46]. East of this current, on the shelf, Arctic Water (ArW) from the east side of Spitsbergen flows north after having rounded South Cape. Thus, the West Spitsbergen Shelf (WSS) is a site where AW and ArW converge forming the Polar Front (PF) [47]. These various water masses create very dynamic ocean conditions along the WSS, which change markedly on a seasonal basis.

During the last decades important climatic changes have occurred in Polar Regions; in this context Svalbard is a hot spot [32]. Heat transported in the AW of the WSC has increased dramatically leading to a general warming of the WSS and adjacent fjords and a dramatic loss of sea ice in the west coast fjords of Svalbard [48]. Such drastic changes are bound to affect the distribution of phytoplankton, zooplankton, fish and hence also top predators. However, predicting future species distributions and status must be based on an understanding of how animals respond to current variation in the environment. Consequently, this study had two aims: i) to characterize the diving/foraging behaviour of harbour seals in Svalbard on a seasonal basis, with a focus on how they deal with High Arctic winter conditions and ii) to assess the oceanographic drivers affecting their behaviour using information on regional hydrography collected by the seals themselves. This will help us understand how harbour seals may be affected by large scale environmental changes in the coming decades and predict their future distribution within the Svalbard Archipelago.

## Materials and Methods

### Animal ethics statement

This study was carried out in strict accordance with the recommendations of the Norwegian Animal Care Authority (Forsøksdyrutvalget) and was approved under permit number 2009/1449. The protocol was also approved by the Governor of Svalbard (Sysselmannen på Svalbard) under permit number 2009/00103-2 a.512 and followed best practice for all animal handling.

### Capturing and tagging

Fifteen juvenile and fifteen adult (total n = 30) harbour seals were live-captured at Forlandøyane on the west coast of Prins Karls Forland (78°20 N, 11°30 E) in the High Arctic Archipelago of Svalbard (Fig 1a) in 2009 and 2010 (n = 15 each year). The animals were captured immediately following their annual moult, between 23 August and 13 September using tangle nets set from shore near haul-out sites (see Lydersen and Kovacs [31] for details). All animals were weighed (Salter spring scales ± 0.5 kg) and sex was determined. Standard length and girth were measured to the nearest cm. Seals were sorted into adult vs juvenile based on a combination of their length, girth and mass measurements following Lydersen and Kovacs [31]. All animals were equipped with Conductivity-Temperature-Depth Satellite-Relay Data Loggers (CTD-SRDLs, Sea Mammal Research Unit (SMRU), University of St Andrews, St Andrews, Scotland http://www.smru.st-andrews.ac.uk/Instrumentation/CTD), glued onto the fur mid-dorsally in the neck area using quick-setting epoxy (tag dimensions 10.5 x 7 x 4 cm, mass 545 g, average 1.0% (range 0.7–1.3%) of seal body mass). All of the seals were also tagged with uniquely numbered plastic tags (Dalton rototags) placed through the webbing of each hind flipper for permanent individual identification.

### CTD-SRDL sampling protocols

Data collected by the CTD-SRDLs (including haul-out and dive behaviour as well as CTD-upcasts [13]) were transmitted via the Argos satellite system (System Argos, Toulouse, France); location estimates of the animals were also calculated by Argos. Accuracies of the CTD data are estimated to be ± 0.02°C for temperature, ± 0.1 mPSU for the derived salinity without correction and 0.3 dBar for pressure [49]. A dive was defined as a period of submersion at least 8 sec long and at least 6 m deep. If either the depth, or the total duration of a dive, was less than these values but the animal was wet, this time was recorded as surface time. If the saltwater switch was dry for more than 10 minutes the time was recorded as haul-out event. The instruments were programmed to send data whenever possible with no duty cycling.

### Data processing

All data processing and analyses were done using the R statistical framework [50]. Satellite-derived locations were first filtered using a speed, distance and angle filter (SDA filter [51]) using the R package “argosfilter” [52]. The swimming speed threshold was set at >2 ms^-1^ and all spikes with angles smaller than 15 or 25 degrees were removed if their lengths were greater than 2.5 or 5 km, respectively. The remaining location estimates were then processed further, using a Kalman filter under a state-space framework using the R package “crawl” [53] that incorporates a covariate for Argos location error (when available) for each of the six Argos location classes (LC—3,2,1,0,A,B). In addition a covariate encompassing the time the animal was hauled out was included allowing the movement along a track-line to stop completely during a haul-out event. Processing the raw location estimates in this manner resulted in a model of the most likely track, from which point location estimates could be interpolated for any specific time. Dive and CTD-cast locations were estimated based on this model using their transmitted time stamps.

### Dive parameters

The dive data stored and transmitted by these instruments fall into two categories: 1) summaries which include the average dive depth, the average dive duration and the total number of dives for each 6 hour-period and 2) a randomly transmitted subset of dives for which a dive profile (based on four inflection points [54]), maximum dive depth and total dive duration are transmitted. Dive parameters (average maximum depth, average duration, number of dives, time spent diving, dive time/surface time) were extracted on a daily basis from the summary data. Only days with a complete record of four summaries were used.

Possible seasonal trends in the dive parameters listed above were explored using Generalized Additive Mixed effect Models (GAMMs). Date was entered as a smoothed term and a separate smoothed term was fitted for each year. Penalised regression splines for date and a Gaussian distribution were used in all the GAMM’s, which were optimised by the restricted maximum likelihood (REML) method. Animal ID was included as a random effect to take into account the pseudoreplication affiliated with multiple points from individual animals and a correlation term was added to take into account the dependence between consecutive days. Possible effects of sex, age, year, ice concentration and interactions year*ice concentration and age*ice concentration were tested. The optimal model was selected using Bayesian Information Criterion (BIC) that penalizes overfitting [55] and BIC weight (BICw). The distribution of the response variables were verified and Gaussian error distributions were used for the GAMMs.

Linear Mixed Effect (LME) models were used to explore the influence of diel periods, year, age, sex and their interactions on the dive depth, dive duration (both log transformed) and number of dives per 6 h period. Sex and diel period and sex and year interactions were only tested for September through December due to the low number of females still transmitting data by January. Similar to the GAMM analyses, animal ID was entered as a random effect and a correlation term was included. Selection of the optimal model was again done using BIC and BICw.

Several metrics were calculated for each of the individually transmitted dives. Bottom time was defined as the time spent at a depth exceeding 80% of the maximum depth reading for a specific dive [56]. However, for deep dives, the transit times to and from the bottom are longer and the amount of time that can be spent at the bottom, within the animal’s physiological constraints, is therefore less than for shallower dives; so bottom time cannot be compared directly between dives of different depths and durations. In order to account for depth and duration of a dive in the bottom time, a multiple regression was fitted between these three parameters following [57] for each individual animal independently. The standardized residuals from this regression were then extracted. Positive residuals indicate dives with a longer bottom time than average for a given depth and duration while negative residuals indicate a shorter bottom time than average, which suggests a decrease in diving effort. In order to identify favoured habitat(s), areas where the animals spent greater amounts of time were identified using the time spent in area (TSA) method following [34]. Areas corresponding to the top 25% of the distribution of TSA for each individual were defined as areas of high usage. Dives located in these areas were extracted, separating them from dives occurring during transit phases and standardized residuals corresponding to these dives in high usage areas were then modelled using GAMMs (see above).

### Environmental data extraction

Daily ice maps constructed by the Norwegian Meteorological Institute were used to determine sea ice concentration categories (http://polarview.met.no/): 1) Open water = 0/10–1/10; 2) Very open drift ice = 1/10–4/10; 3) Open drift ice = 4/10–7/10; 4) Close drift ice = 7/10–9/10; 5) Very close drift ice = 9/10–10/10 and; 6) Land fast ice (ice that makes contact with shore). Ice concentration was extracted at the estimated geographical location of each dive using the R package “Raster” [58].

Bathymetry was extracted for each dive location from a 0.5 km x 0.5 km resolution data set from the International Bathymetric Chart of the Arctic Ocean (IBCAO version 3.0, 2012, http://www.ibcao.org [59]). Dive types were assigned using the definition from [60] with respect to bathymetry. Dives occurring in waters shallower than 50 m were classified as “coastal” and dives in waters deeper than 50 m were classified according to what part of the water column was used (dive depth divided by bathymetric depth). If the ratio was >0.95 the dive was classified as “benthic” and if the ratio was <0.95 it was classified as “pelagic”. A multinomial model was fitted to explore the relationship between the dive type, month, year and age. Selection of the optimal model was done using BIC.

Visualization of oceanographic data was done using Ocean Data View (ODV) 4.5.1 [61] using raw data sent by the tag. No post-processing of the CTD data was performed because the precision of these data were sufficient for comparing characteristics of known water masses (see [43] for details). Individual points belonging to each CTD cast collected by all the individuals within a geographical region were interpolated using the Data-Interpolation Variational Analysis (DIVA) gridding method in order to create isosurfaces of the different variables [62].

Temperature and salinity were extracted for each dive at the surface (i.e. 6 m corresponding to the first data point within a dive) and at the maximum depth using information collected by the CTD-SRDLs. CTD profiles were not available for every dive and the daily number of casts per animal varied greatly (Table 1). However, all of the animals stayed within a limited geographical region on the shelf (Fig 1b) so temperature, salinity and distance to the mixed layer depth (see below) could be ascribed to each dive using the entire CTD dataset. An interpolation method based on a weighted running average using the distance and time between a dive and the neighbouring CTD casts was implemented. The weights were based on a standardized Gaussian distribution with a standard deviation of three days and 25 km for the time and distance respectively with the mean of the distribution falling at the time of the dive of interest and its location. In addition, a vertical weighting with a standard deviation of 5 m (twice the sensor resolution) was used so that each temperature and salinity value was a result of an interpolation incorporating 3D spatial and time dimensions.

**Table 1:** Summary statistics for harbour seal dives and CTD casts collected by animal borne CTD-SRDLs.

		Dives										CTD casts			
Year	Seal ID	AvDepth	SDdepth	MaxDepth	AvDur	SDdur	MaxDur	AvBott	SDbott	MaxBott	% transm	Tot. nb. Casts	Nb. casts/day	AvDepth	SDdepth
2009	F41	19	19	186	137	77	1020	86	61	433	26.0	183	0.7	50	43
2009	F44	35	27	148	161	72	780	110	54	413	21.5	206	1.4	50	29
2009	F47	15	8	90	116	55	780	79	51	368	31.1	53	0.4	29	43
2009	F48	10	3	33	96	38	300	56	35	211	30.5	4	0.0	20	8
2009	F60	23	14	176	167	64	750	124	62	456	21.8	284	1.4	30	30
2009	F66	15	10	85	141	58	2235	98	57	395	19.6	61	0.6	22	14
2009	F74	13	8	110	149	74	570	97	61	425	19.0	28	0.2	27	20
2009	F76	41	32	241	285	101	870	219	105	672	30.9	661	2.4	36	34
2009	M43	37	23	186	168	79	600	112	67	467	24.1	382	2.2	44	33
2009	M51	72	51	316	258	121	1020	152	85	802	35.2	716	2.6	88	58
2009	M52	61	36	271	223	90	660	148	75	605	29.3	683	2.5	65	42
2009	M56	70	55	301	231	115	1740	134	83	859	28.8	526	1.9	98	60
2009	M64	58	45	296	231	107	1020	150	82	774	29.7	587	2.3	74	56
2009	M65	56	39	326	239	99	930	159	87	641	24.2	593	2.2	69	48
2009	M77	17	12	100	140	66	570	91	58	429	22.7	62	0.6	32	17
2010	F42	13	4	24	153	56	405	122	59	379	3.8	21	0.1	29	17
2010	F44	25	19	283	187	55	425	128	59	403	6.0	314	2.2	33	20
2010	F50^a^	11	4	26	132	32	245	98	43	222	4.4	1	0.0	NA	NA
2010	F53	46	34	189	260	128	855	187	99	642	6.0	560	3.6	51	36
2010	F58a	34	23	177	178	61	775	119	52	385	4.8	285	3.4	48	32
2010	F58b	79	62	299	309	152	1875	181	114	664	9.1	1325	4.5	115	59
2010	F59	21	13	81	195	61	495	160	59	395	3.7	204	2.0	24	14
2010	M41	73	55	331	235	104	775	122	71	491	6.6	1427	5.0	94	66
2010	M45	16	10	69	127	58	385	79	51	367	3.3	94	0.6	40	31
2010	M48	42	29	283	240	93	775	171	83	511	6.8	1156	4.0	44	40
2010	M53a	58	38	387	270	111	2235	187	94	800	8.1	1421	4.9	59	43
2010	M53b	74	57	403	242	127	975	125	77	496	7.7	846	4.8	113	67
2010	M57	57	41	307	267	109	1475	192	92	765	8.5	1350	4.5	58	44
2010	M64	92	61	363	301	133	935	167	92	613	10.4	1482	5.1	124	73
2010	M65	44	32	195	317	120	935	234	114	709	10.0	1127	4.8	41	37

The information presented is for 30 adult and juvenile harbour seals equipped with Conductivity-Temperature-Depth Satellite-Relay-Data-Loggers (CTD-SRDLs) in Svalbard, Norway during 2009/2010 and 2010/2011. *AvDepth* average depth (m), *AvDur* average duration (sec), *AvBott*, average time spent at the bottom of the dive (sec) during the tagging period; *MaxDepth* maximum depth (m), *MaxDur* maximum duration (sec), *MaxBott* maximum time spent at the bottom of the dive (sec), *SDdepth* standard deviation for depth (m), *SDdur s*tandard deviation duration (sec), *SDbott s*tandard deviation time spent at the bottom of the dive (sec) during the tagging period. *% transm* represents the percentage of dives that were transmitted during the tagging period. Seal ID contains information on the sex and weight. If two or more animals had the same sex and weight and were tagged the same year, alphabetic indices were used (ex: F58a F58b).

^a^ Only one cast was collected by this instrument and it was therefore not used in the analysis.

Three geographical regions were defined where most of the CTD casts were collected by the seals in order to construct meaningful time series for temperature and salinity between the two years of the study: box A in the northwest corner of Spitsbergen; box B west of PKF and; box C south of PKF (Fig 1c and 1d). This focussed the analyses on relatively homogenous oceanographic regions, essentially removing the few points collected in fjords, beyond the shelf edge and between PKF and Spitsbergen, which all belong to different oceanographic domains. Temperature data points were smoothed using a moving Gaussian weighted average with a window of 2 days.

Analyses within the water column were conducted to determine the depths at which water masses changed, so that seal diving could be linked to specific (or mixed) water masses. The mixed layer is defined as a surface layer where there is nearly no variation in density with depth, i.e. the layer represents a quasi-homogeneous region [63]. The maximum depth of this layer was calculated based on the density profiles method, using a sharp change in the relationship between seawater potential density and depth as being indicative of the change to another water mass [63]. The sea water potential density was determined for the first data point in a dive (6 m) in order to avoid turbulence inherent in surface processes such as wave action. It was calculated according to McDougall et al. [64] using the R package “OCE” [65].

### Linking oceanographic and dive data

To explore the influence of dynamic oceanographic features such as the mixed layer depth on dive behaviour, daily differences between dive depth and the mixed layer depth were calculated. As done previously, only dives occurring over the shelf were used, not those few that occurred beyond the shelf edge or in the fjords. The difference in depth between the bottom of the mixed layer and the daily average dive depth was calculated and modelled using Generalized Additive Models (GAMs) including an interaction between the date (entered as a smooth term) and the year of tagging. Change point analysis was used to determine the date at which a significant change in the difference in depth occurred [66].

Coastal upwelling can be wind-induced through Ekman drift [67]. This phenomenon has been described recently for the WSS, where northerly/northeasterly winds cause cross-shelf exchanges [46,47,68]. Therefore wind stress from the north or north-east was used as a proxy for upwelling. The influence of wind speed was tested on average daily dive parameters (dive duration, dive depth, time spent at the bottom and temperature and salinity at the maximum dive depth). Average wind speed from the north or combined north and east directions preceding the dive was used as a covariate for the GAMs. Several time lags were explored (1–7 days). Date and wind speed were entered as smooth terms with an interaction with the year of tagging. Model selection was done by BIC. Wind stress data were obtained from the Norwegian Meteorological Institute (www.met.no) for a grid point at 78.5° N and 12.0° N (West of PKF), which has been shown by correlation studies to give a good representation of the wind stress field over the WSS [68,69].

## Results

The 30 CTD-SRDLs deployed on adult and juvenile harbour seals in this study provided data for periods ranging from 54 to 298 days with an average data record lasting 200 ± 79 days (S1 Table). Twelve animals had records that extended into June the year following tag deployment; but all tags ceased to function before July. Year of tag deployment (ANOVA F_1,58_ = 2.14, p = 0.15) and seal maturity class (ANOVA F_1,58_ = 0.92, p = 0.40) had no effect on the duration of the tracking records; but males had longer data records than females in both years (ANOVA F_1,58_ = 6.62, p<0.01). The percentage of transmitted dives varied greatly between individuals (range 3.3%- 35.2%) and between years (mean ± SD) (mean _2009_ = 26.3% ± 4.8; mean _2010_ = 6.6% ± 2.3) due to a different sampling protocol for dives and CTD casts in the two consecutive seasons (Table 1). Similarly, the number of CTD casts per day varied between individuals depending on their diving behaviour (range 0.1–5.1 casts/day) and between years (mean _2009_ = 1.4 casts/day ± 0.9; mean _2010_ = 3.3 casts/day ± 1.8) (Table 1). One instrument collected only 1 cast (F50-10) over a 54 day period so the information from this cast was not used in subsequent analyses, but dives belonging to this individual were assigned oceanographic information (temperature and salinity) based on information collected by neighbouring individuals.

A total of 189,104 dives (143,168 in 2009/2010 and 40,736 in 2010/2011) were recorded by the 30 instrumented seals during the two years of the study. Individual animals dove to a mean depth of 41 m ± 24 (SD) with a maximum dive depth range of 24–403 m. Dives lasted on average 204 s ± 121 with maximum durations ranging between 245–2,235 s (Table 1). A total of 5,028 CTD casts (mean depth 63 ± 51 m) were collected in 2009/2010 and 11,612 (mean depth 76 ± 62 m) in 2010/2011.

**Fig 2:**
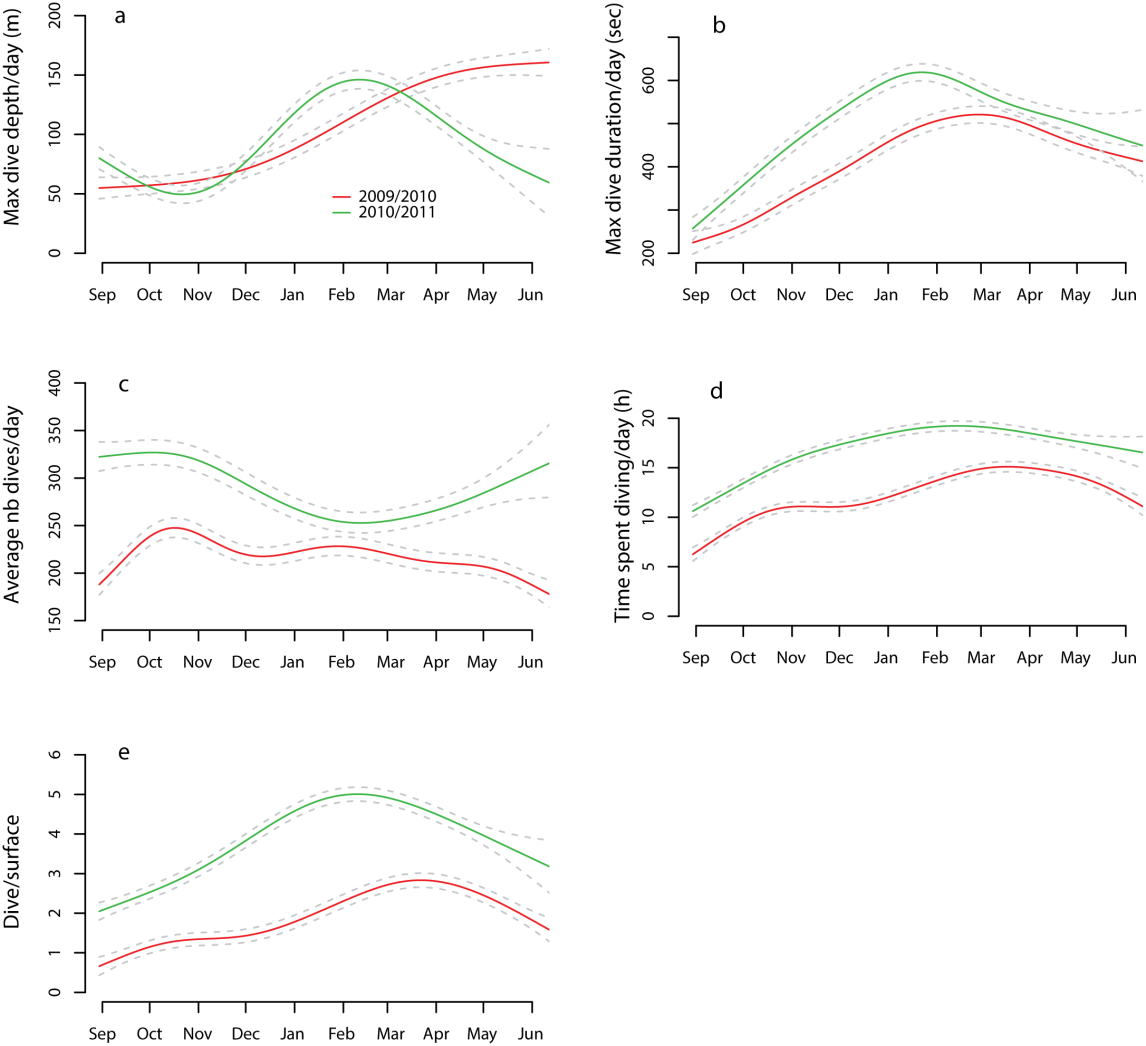
Mean daily dive parameters as a function of date. Predicted GAMM curves (solid lines) ± 95% CI (dotted lines) for (a) maximum dive depth (b) maximum dive duration (c) average number of dives (d) time spent diving (e) and dive/surface time as a function of the date for 30 adult and juvenile harbour seals equipped with Conductivity-Temperature-Depth Satellite-Relay-Data-Loggers (CTD-SRDLs) in Svalbard, Norway during 2009/2010 and 2010/2011. The model is comprised of a random term (animal ID), a different smooth term fitted for each year and a first order correlation term (corAR1).

All average daily dive parameters analysed (depth, duration, diving time and dive time/surface time) as well as the total number of dives per day were influenced by date, while sex, age, sea-ice concentration and their interactions were not particularly influential (Table 2). Daily maximum dive depth, dive duration, time spent diving and the dive/surface ratio increased through the winter with a marked maximum for 2010/2011 occurring in January/February. The maximum was less pronounced in 2009/2010 and occurred later in the season (March/April) (Fig 2). The total number of dives per day followed an inverse trend compared to the other dive parameters, decreasing during the winter and early spring in both years.

**Table 2:** Selection table for Generalised Additive Mixed Models exploring the variation in dive parameters and activity budget.

Response variable	Model structure	BIC	BICw	Δ BIC
**Max depth/ day**	***f(date*,*by=year)*, *cor=corAR1()*, *random=1|ID***	**39514.56**	**0.47**	0.00
family=gaussian	*f(date*,*by=year)+sex*, *cor=corAR1()*, *random=1|ID*	39514.77	0.42	0.21
	*f(date*,*by=year)+year*, *cor=corAR1()*, *random=1|ID*	39519.18	0.05	4.62
	*f(date*,*by=year)+sex+year*, *cor=corAR1()*, *random=1|ID*	39519.19	0.05	4.64
	*f(date*,*by=year)+age*, *cor=corAR1()*, *random=1|ID*	39522.79	0.01	8.23
	*f(date*,*by=year)+age+sex*, *cor=corAR1()*, *random=1|ID*	39523.02	0.01	8.47
**Max dive duration/day**	***f(date*,*by=year)+year*, *cor=corAR1()*, *random=1|ID***	**52247.11**	**0.80**	0.00
family=gaussian	*f(date*,*by=year)+year+age*, *cor=corAR1()*, *random=1|ID*	52251.53	0.09	4.42
	*f(date*,*by=year)*, *cor=corAR1()*, *random=1|ID*	52252.14	0.06	5.03
	*f(date*,*by=year)+sex+year*, *cor=corAR1()*, *random=1|ID*	52253.03	0.04	5.92
	*f(date*,*by=year)+sex+year+age*, *cor=corAR1()*, *random=1|ID*	52256.77	0.01	9.66
**Average Nb dives/day**	***f(date*,*by=year)+year*, *cor=corAR1()*, *random=1|ID***	**581.02**	**0.89**	**0.00**
family=poisson	*f(date*,*by=year)+sex+year*, *cor=corAR1()*, *random=1|ID*	586.39	0.06	5.38
	*f(date*,*by=year)*, *cor=corAR1()*, *random=1|ID*	587.66	0.03	6.64
	*f(date*,*by=year)+year+age*, *cor=corAR1()*, *random=1|ID*	589.28	0.01	8.26
**Average time spent diving/day**	***f(date*,*by=year)+year*, *cor=corAR1()*, *random=1|ID***	**581.02**	**0.89**	**0.00**
family=gaussian	*f(date*,*by=year)+sex+year*, *cor=corAR1()*, *random=1|ID*	586.39	0.06	5.38
	*f(date*,*by=year)*, *cor=corAR1()*, *random=1|ID*	587.66	0.03	6.64
	*f(date*,*by=year)+year+age*, *cor=corAR1()*, *random=1|ID*	589.28	0.01	8.26
**Dive/surface**	***f(date*,*by=year)+year*, *cor=corAR1()*, *random=1|ID***	**11889.82**	**0.80**	0.00
family=gaussian	*f(date*,*by=year)+year+age*, *cor=corAR1()*, *random=1|ID*	11893.32	0.14	3.50
	*f(date*,*by=year)+sex+year*, *cor=corAR1()*, *random=1|ID*	11895.51	0.05	5.69
	*f(date*,*by=year)+sex+year+age*, *cor=corAR1()*, *random=1|ID*	11899.08	0.01	9.26

The models presented best fitted daily maximum dive depth, number of dives, time spent diving, dive duration and dive/surface for 30 adult and juvenile harbour seals equipped with Conductivity-Temperature-Depth Satellite-Relay-Data-Loggers (CTD-SRDLs) in Svalbard, Norway during 2009/2010 and 2010/2011. Each model was fitted with a random (animal ID) and a first-order autocorrelation term (corAR1). Only models with a BICw ≥0.01 are presented and were selected using BIC. Top-ranked models are in bold.

**Fig 3:**
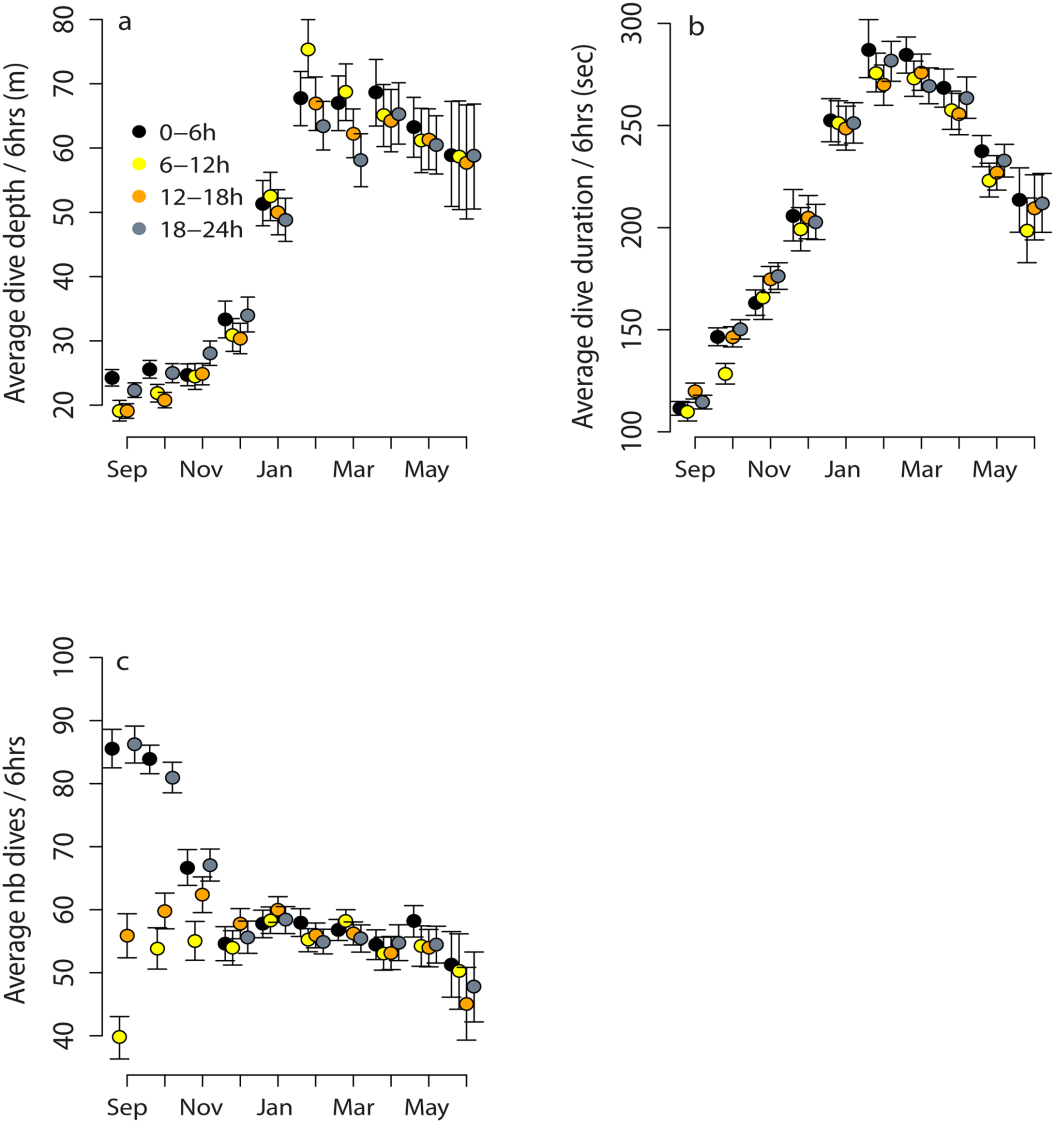
Influence of the time of day on dive parameters as a function of the season. Mean and 95% bootstrapped CI of (a) average dive depth (b) average dive duration and (c) average number of dives per 6 hour period for each month made by harbour seals instrumented with Conductivity-Temperature-Depth Satellite-Relay-Data-Loggers (CTD-SRDLs) on Svalbard, Norway during 2009/2010 and 2010/2011.

Diel patterns were apparent in dive duration and depth during the autumn (Fig 3, Table 3). Dives that occurred during the night (0–6 h and 18–24 h) were deeper, slightly longer and more numerous than during daytime (6–18 h). This diel pattern disappeared during the Polar Night and did not reappear during spring or early summer. A diel period/year interaction was significant in a few months but this was mainly because animals dove slightly longer and deeper in the second year of the study. In both years, diel period had similar influences on dive parameters, with the greatest impact on the number of dives performed (Fig 3).

**Table 3:** Structure of LME models exploring the influence of diel pattern.

Response variable	Month	Model structure	BIC	weight
Dive depth	September	period	4131.03	0.85
	October	period+sex+period*sex	4961.57	0.91
	November	Null model	4519.58	0.71
	December	Null model	4473.66	0.85
	January	Null model	4267.91	0.77
	February	year	3187.50	1.00
	March	Null model	3233.45	0.57
	April	Null model	3192.80	1.00
	May	Null model	2922.38	0.70
	June	Null model	1067.21	0.53
Dive duration	September	period+year+period*year	2178.73	0.90
	October	period	2513.37	0.58
	November	period	2506.07	0.52
	December	year	2894.42	0.74
	January	year	2503.54	0.84
	February	year	1409.69	0.65
	March	Null model	1066.85	0.87
	April	Null model	1650.88	0.93
	May	year	1264.78	0.49
	June	year	598.65	0.48
Number of dives	September	period+year+period*year	23938.44	0.88
	October	period+year	23533.71	1.00
	November	period+year	20522.43	0.95
	December	Null model	18989.04	0.75
	January	Null model	19114.79	1.00
	February	Null model	14392.01	1.00
	March	Null model	12830.28	0.93
	April	Null model	11284.07	0.91
	May	year	10596.32	1.00
	June	year	4162.48	0.94

BIC and BIC weight of Linear Mixed Effect models (LME’s) which best fitted average dive depth, duration and number of dives per 6 hour periods within each month for 30 adult and juvenile harbour seals equipped with Conductivity-Temperature-Depth Satellite-Relay-Data-Loggers (CTD-SRDLs) in Svalbard, Norway during 2009/2010 and 2010/2011 are presented. Each model was fitted with a random (animal ID) and a first-order autocorrelation term (corAR1).

**Fig 4:**
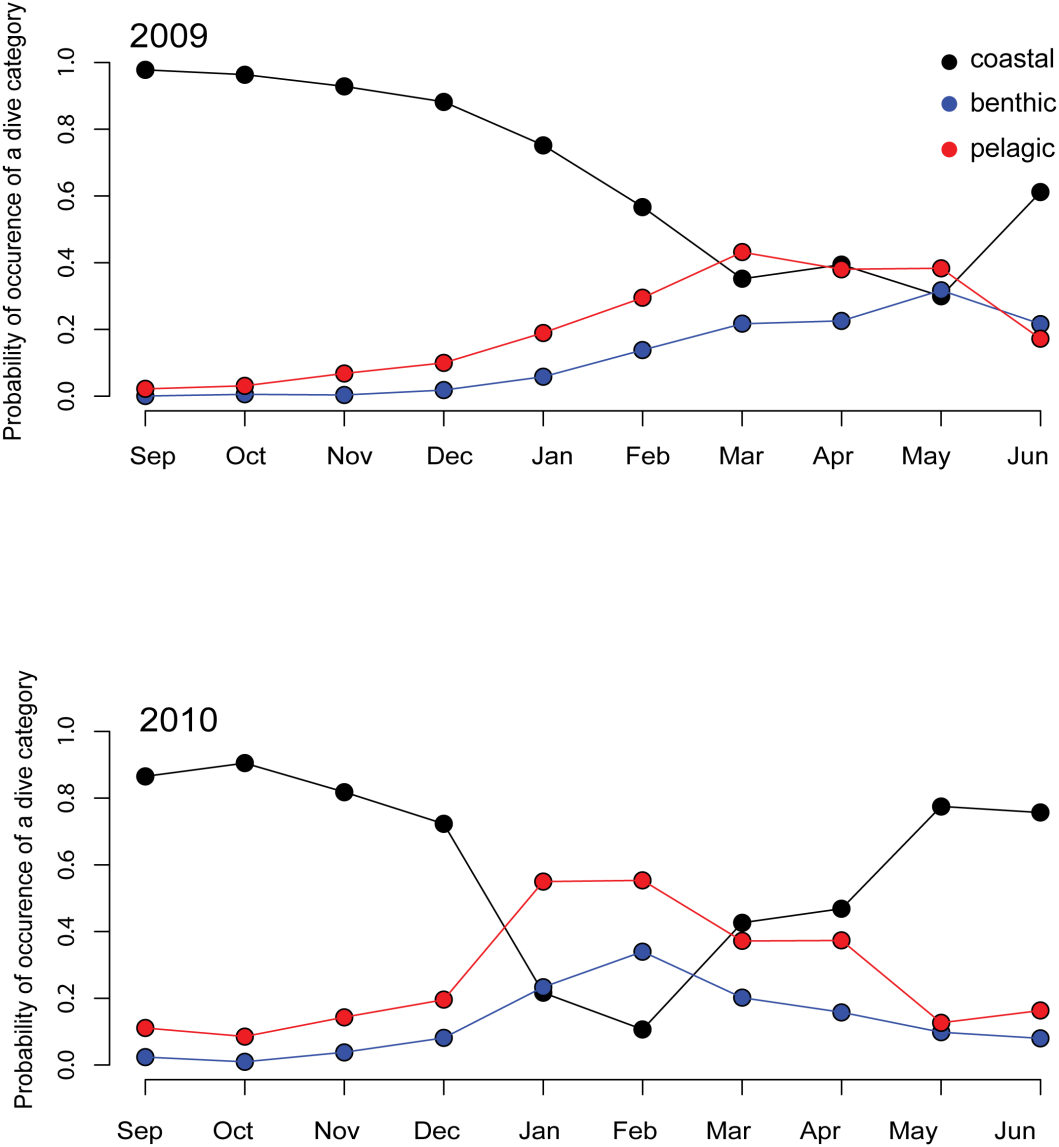
Dive type as a function of the date. Predicted probabilities of belonging to a dive category with respect to bathymetry (coastal, benthic, pelagic) based on the optimal selected multinomial model (dive category ~ month*year) for 30 adult and juvenile harbour seals equipped with Conductivity-Temperature-Depth Satellite-Relay-Data-Loggers (CTD-SRDLs) in Svalbard, Norway during 2009/2010 and 2010/2011.

Sixty-eight percent of the total number of transmitted dives were performed in coastal waters (<50 m) while the remaining 32% occurred in water of greater depth. Of this 32%, 21% were classified as pelagic and 11% as benthic. The proportion of dives in the various categories varied by month, with the proportion of coastal dives decreasing through the winter (Fig 4) and the proportion of benthic dives increased slightly through the winter, while pelagic dives were most numerous from March through May in 2009/2010 and from January through April in 2010/2011. The optimal multinomial model included month*year (S2 Table) showing an influence of the year of tagging on the calendar month.

**Fig 5:**
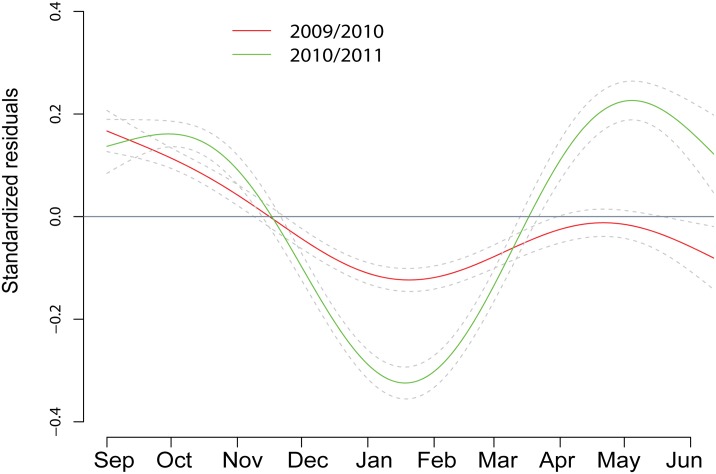
Seasonal pattern of foraging effort. Predicted GAMM curves (mean (solid lines) and ± 95% CI (dotted lines)) for foraging effort by 30 adult and juvenile harbour seals equipped with Conductivity-Temperature-Depth Satellite-Relay-Data-Loggers (CTD-SRDLs) on Svalbard, Norway during 2009/2010 and 2010/201. Foraging effort is defined as the bottom time corrected for depth and duration and is represented by the standardized residuals resulting from the multiple regression (bottom time ~ dive depth + dive duration). The dives used in this analysis are located in areas of high usage (defined as dives located in areas where individuals spent most of their time). The model is comprised of a random term (animal ID), a first order correlation term (corAR1) and a different smooth term is fitted for each year.

There was a significant seasonal change in diving effort for dives located in areas of high use (Fig 5). Diving effort was influenced by date and year of tagging, while age and sex were not retained in the optimal model linking these parameters (Table 4). Animals spent a smaller amount of time at the bottom of a dive with respect to depth and duration in winter compared to early autumn. This seasonal variation was more evident in 2010/2011 than in 2009/2010.

**Table 4:** Generalised Additive Mixed Models exploring the variations in diving effort.

Model structure	BIC	BICw	Δ BIC
*f(date*,*by=year)*, *cor=corAR1()*, *random=1|ID*	60872	0.98	0.46
*f(date*,*by=year)+age*, *cor=corAR1()*, *random=1|ID*	60881	0.01	9.39
*f(date*,*by=year)+sex*, *cor=corAR1()*, *random=1|ID*	60882	0.01	10.46
*f(date*,*by=year)+sex+age*, *cor=corAR1()*, *random=1|ID*	60891	0.00	19.36

Candidate models which best fitted the standardized residuals obtained from the multiple linear regression (bottom time~ dive depth + dive duration) for 30 adult and juvenile harbour seals equipped with Conductivity-Temperature-Depth Satellite-Relay-Data-Loggers (CTD-SRDLs) in Svalbard, Norway during 2009/2010 and 2010/2011. Each model was fitted with a random (animal ID) and a first-order autocorrelation term (corAR1()) and the best fitted model was selected using BIC. Top-ranked models are in bold.

**Fig 6:**
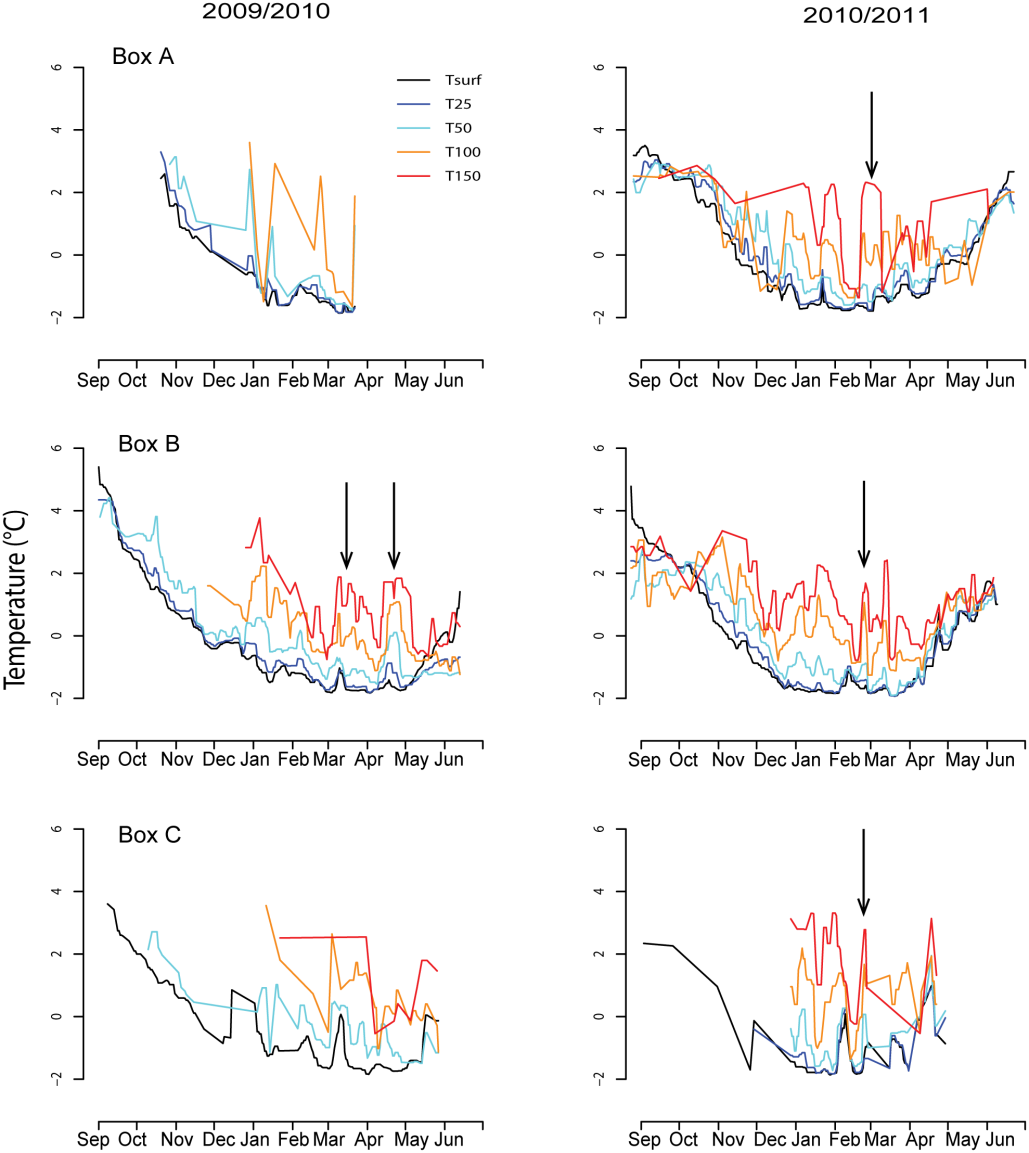
Temperature according to depth and date for three geographical regions. Weighted average daily temperature from the surface to 150 m depth for both study years (left 2009/2010; right 2010/2011) within each geographical region (see Fig 1). The black arrows show the most prominent upwelling events. Note that there was no measurement at 150 m in Box A during the first season.

**Fig 7:**
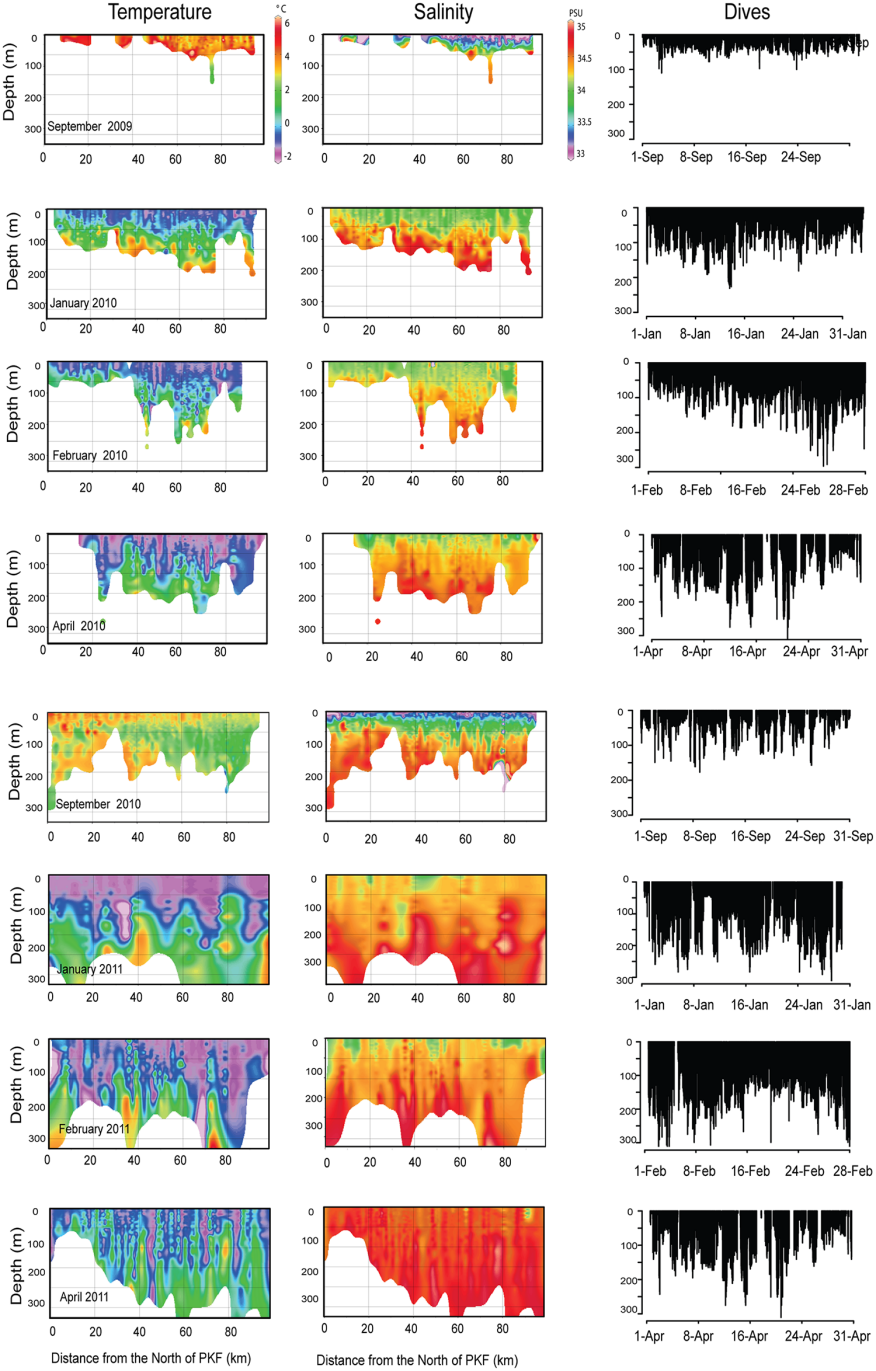
Temporal changes in temperature and salinity in the water column on the West coast of Prins Karl Forland (PKF) derived from CTD profile data. Temperature and salinity profiles show a vertical stratification occurring during the winter months with warmer and more saline water occupying the bottom part of the water column. The dive panels on the right represent the vertical profiles of dives occurring in the correspondent area during the same month. Only selected months are presented to reduce the number of Figures, while still capturing the temporal oceanographic variability.

The warmest water temperatures were documented in September in both years (2°- 5°C), within the surface layers. These layers cooled progressively and reached a minimum (- 1.9°C) in February—March (Fig 6). However, at greater depths (≥100 m) surges of warmer water (between 3°C and 4.8°C) occurred during winter, from December until April in both years. These influxes of warmer water occurred at depths between 100 and 150 m, but they had influence throughout the entire water column up to the surface. Temperatures greater than 3.5°C at depths ≥ 100 m were only recorded in January—February in both years. Salinity changed concomitantly with temperature (Fig 7). The influxes of warmer, more saline AW occurred over the entire study area, covering the WSS (S1 Fig).

**Fig 8:**
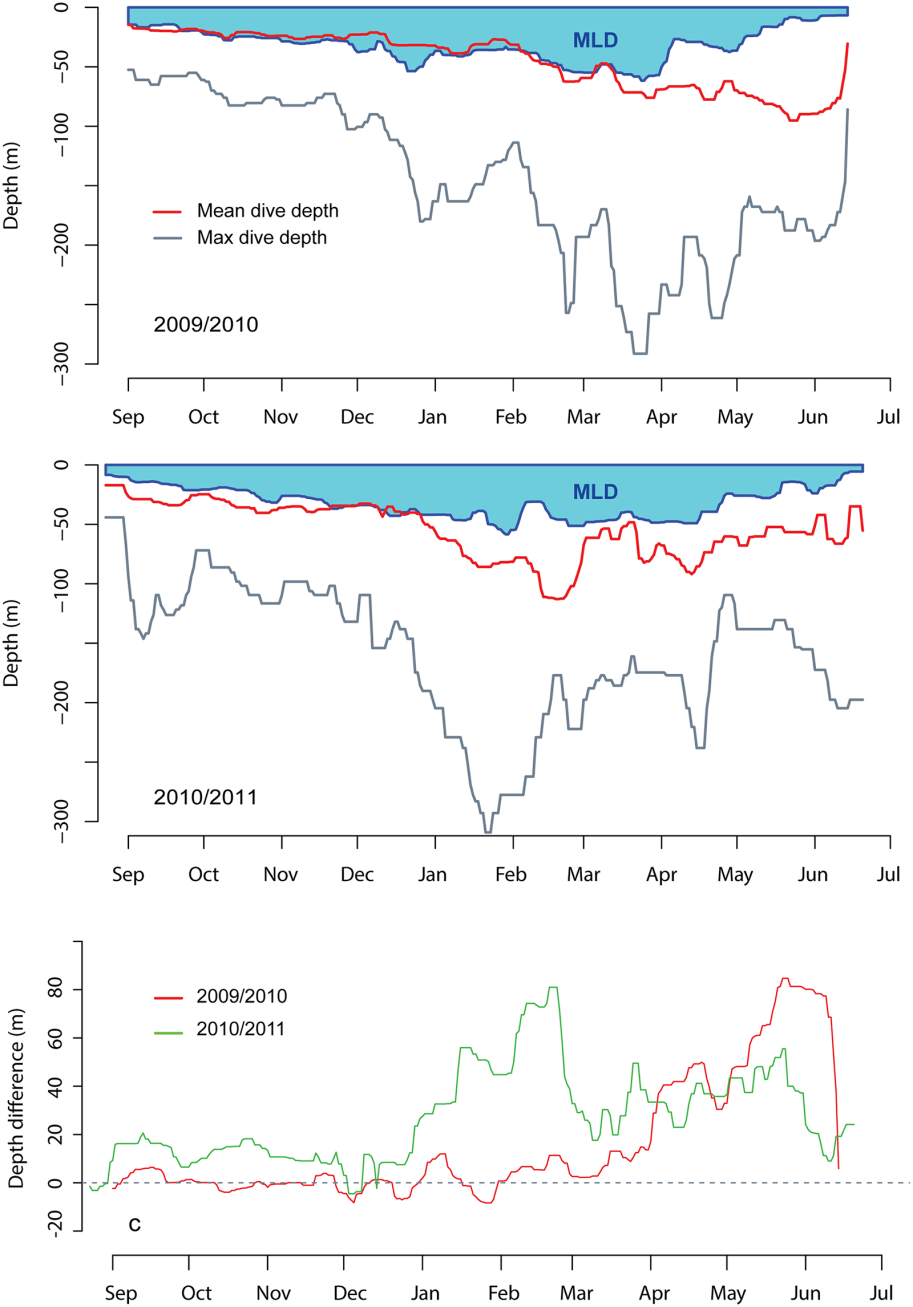
Diving depth in relation to the mixed layer depth (MLD). Depth of the mixed layer (in blue), daily average dive depth (in red) and daily maximum dive depth (in grey) as a function if the date for harbour seals instrumented with Conductivity-Temperature-Depth Satellite-Relay-Data-Loggers (CTD-SRDLs) in Svalbard, Norway during 2009/2010 and 2010/2011. All of the dives presented were located over the shelf. The bottom panel represents the difference in depth between the MLD and the average dive depth for both study seasons. All curves are smoothed with a moving, weighted mean which has a window of 3 days.

There was a strong relationship between mixed layer depth, calendar month, year of tagging and the average daily dive depths performed by the seals over the shelf (Fig 8). The difference between the depth of the mixed layer and the average daily dive depth was dependant on date and year (GAM - F_s(date, 2009)_ = 72.11 p< 0.001; F_s(date,2010)_ = 26.45 p< 0.001). However, individuals dove consistently down to the MLD during the autumn and early winter during both years of the study. Change point analysis showed that a deviation from the mean occurred first on the 3^rd^ of April 2010 in the first year of the study and on the 27^th^ of December 2010 for the second year of the study. After these dates, the seals started diving deeper than the MLD.

There were positive relationships between weekly-averaged northerly or north-easterly wind stress and dive parameters (Table 5), most influentially in the model when using the average wind speed the week before a dive. Temperature and salinity at the bottom of a dive and mean dive duration increased with wind stress from northerly directions. Three competing models with Δ BIC < 1 were selected to explore the relationship between mean dive depth and wind stress. Two of them included average north-easterly wind stress (Table 5). Interaction between wind stress and the year of tagging was only significant for temperature at the bottom of the dive.

**Table 5:** Generalised Additive Mixed Models exploring the variations in diving parameters.

Response variable	Model structure	BIC	BICw	Δ BIC	df
Tmax	***f(date*,*by=year)+f(North*,*by=year)***	**1407.64**	**0.96**	0.00	**16.90**
	*f(date*,*by=year)+ North*	1415.26	0.02	7.62	10.03
	*f(date*,*by=year)+f(North)*	1415.31	0.02	7.67	16.13
	*f(date*,*by=year)*	1431.53	0.00	23.89	9.31
	*f(date*,*by=year)+ NorthEast*	1434.35	0.00	26.71	10.30
	*f(date*,*by=year)+f(NorthEast*,*by=year)*	1439.78	0.00	32.14	13.95
	*f(date*,*by=year)+f(NorthEast)*	1442.13	0.00	34.48	14.92
Smax	***f(date*,*by=year)+ North***	**792.50**	**0.45**	0.00	**9.34**
	***f(date*,*by=year)***	**792.90**	**0.37**	0.41	**8.34**
	*f(date*,*by=year)+f(North)*	795.11	0.12	2.62	15.00
	*f(date*,*by=year)+ NorthEast*	798.00	0.03	5.50	9.43
	*f(date*,*by=year)+f(NorthEast)*	798.00	0.03	5.50	9.43
	*f(date*,*by=year)+f(North*,*by=year)*	806.92	0.00	14.42	13.37
	*f(date*,*by=year)+f(NorthEast*,*by=year)*	816.68	0.00	24.19	15.98
Mean dive depth	***f(date*,*by=year)***	**4790.60**	**0.36**	0.00	**9.67**
	***f(date*,*by=year)+ NorthEast***	**4790.75**	**0.34**	0.15	**10.71**
	***f(date*,*by=year)+f(NorthEast)***	**4791.29**	**0.26**	0.68	**10.84**
	*f(date*,*by=year)+f(NorthEast*,*by=year)*	4795.98	0.02	5.37	11.73
	*f(date*,*by=year)+ North*	4796.54	0.02	5.94	10.64
	*f(date*,*by=year)+f(North*,*by=year)*	4801.05	0.00	10.45	14.38
	*f(date*,*by=year)+f(North)*	4820.59	0.00	29.99	17.85
Mean dive duration	***f(date*,*by=year)+ North***	**5796.55**	**0.92**	0.00	**10.33**
	*f(date*,*by=year)*	5802.05	0.06	5.50	9.39
	*f(date*,*by=year)+f(North*,*by=year)*	5804.90	0.01	8.35	13.17
	*f(date*,*by=year)+ NorthEast*	5806.69	0.01	10.14	10.37
	*f(date*,*by=year)+f(NorthEast)*	5813.99	0.00	17.44	14.53
	*f(date*,*by=year)+f(NorthEast*,*by=year)*	5814.23	0.00	17.67	11.79
	*f(date*,*by=year)+f(North)*	5816.17	0.00	19.61	16.99

Generalised Additive Models which best fitted (estimated using Δ BIC) daily temperature at the bottom of the dive (Tmax), salinity at the bottom of a dive (Smax), maximum dive depth and dive duration as a function of the date and averaged weekly northerly (North) or north-easterly (NorthEast) winds the precedent week for 30 adult and juvenile harbour seals equipped with Conductivity-Temperature-Depth Satellite-Relay-Data-Loggers (CTD-SRDLs) in Svalbard, Norway during 2009/2010 and 2010/2011. Top-ranked models are in bold.

## Discussion

Harbour seals occur throughout the North Pacific and North Atlantic and thus occur in a wide variety of habitats, leading to high levels of variation in behaviour at local or regional scales [70], rendering it challenging to identify general species-specific patterns. In addition, this species shows high site fidelity and regionally specific prey utilisation [71,72] which pose a challenge in terms of global assessment of potential impacts of climate change [73]. Identifying population-specific patterns in relation to environmental conditions is therefore crucial. This study presents novel telemetry data spanning from the early fall (post-moulting) to the following breeding season in early summer, in combination with small-scale hydrographic data collected by the seals themselves, allowing for exploration of water masses in relation to quite fine-scale movements for two successive years. Despite significant inter annual variation, this extensive dataset permitted the exploration of small-scale variation in diving behaviour throughout a seasonal range of environmental conditions in the most extreme conditions experienced by this species, providing significant insight into the behavioural plasticity and likely responses to climate change by this species.

Harbour seals are shallow, coastal divers with individuals normally using only the top 50 m of the water for dives of a few minutes duration, within 50 km of their haul-out sites [34,56,71,72,74]. However, according to local prey availability, bathymetry, bottom sediment types and local hydrographic conditions, dives parameters are variable [40,72]. Only two earlier studies have focused on diving behaviour of adult and juvenile harbour seals from the Svalbard population; Gjertz et al. [30] deployed 14 SRDLs and Krafft et al. [29] report records from 3 Time Depth Recorders. Both of these studies were shorter than the present study and focussed solely or mainly on the immediate post-moulting period. In the present study, dives were shallow (50% of the dives shallower than 30 m) and short (50% of the dives shorter than 3 min) similar to dive parameters reported by Gjertz et al. [30], but both average depth and duration were greater than those reported in Krafft et al. [29], likely because the latter study included only data collected in September for three juvenile females.

Harbour seals generally display strong seasonal patterns to their annual behavioural/physiological cycles [18,75]. They haulout onto solid substrates regularly and for long periods during the breeding and moulting seasons in summer and early autumn. During these times they depend heavily on their body reserves and thus lose mass. The period of intense feeding that follows the moult is vital to replenish their body stores before the winter [18]. In the present study, diving behaviour changed markedly on a seasonal basis, with several dive metrics showing concomitant changes which are likely linked to seasonal changes in prey distribution and type [70,76–78] as well as changes in the seals’ energy requirements [79]. In the autumn, animals dove to moderate depths (less than 50 m) for moderate durations (ca 3 min), but did so rather intensively with a maximum number of dives/day occurring in October for both study years (Fig 2). Animals also spent more time at the bottom of their dives at this time compared to the annual average (Fig 5), again showing intense diving effort. Most of the diving in this period of the year occurred during the night.

Across their range, harbour seals consume a wide variety of prey including invertebrates and both benthic and pelagic fish. In Svalbard fish dominate the diet, but squid, crustaceans and polychaetes are also eaten in small quantities [43,44]. In the autumn Atlantic cod *(Gadus morhua)*, and polar cod *(Boreogadus saida)*, followed by shorthorn sculpin (*Myoxocephalus scorpius)* are the main prey items of these harbour seals [43,44]. Atlantic cod concentrates around the PF in both benthic and pelagic habitats from the surface down the bottom (down to 600 m as the water gets deeper westward over the shelf slope; (see [80] for details) in the vicinity where all of the instrumented harbour seals remained after the moult. Although Atlantic cod is a largely temperate species, it is increasingly common on the WSS in Svalbard due to an increase in the influx of AW through the WSC [68,81–83]. Atlantic cod and haddock *(Melanogrammus aeglefinus)* tend to stay at depths close to the seabed during the day and move upwards in the water column during the night, where they become more accessible to the seals [84,85]. This is consistent with the observed diel patterns in the seal’s diving patterns; dives are more numerous at night. Polar cod is considered to be a semi-pelagic species that exhibits size/depth segregation, but it is mainly linked to ArW masses [86]. Large schools have been observed in shallow water (<10 m) during the autumn in northern Canada [85], and also in late summer in Svalbard (pers. obs.) which could make shallow foraging profitable for the seals at this time of the year. Polar cod, similar to Atlantic cod, undertake diel vertical migrations during the period of the year that has light/dark cycling [83,85].

In the winter, dives performed by the harbour seals in Svalbard were longer, deeper and less numerous. The total time spent diving per day and the ratio of dive time to surface time increased (Fig 2), while the foraging effort within individual dives decreased (seals spent less time at the bottom of dives when correcting for depth and duration) (Fig 5). Gjertz et al. [30] have previously shown that dives were longer and deeper in December through February compared to October for this population. Similar observations of seasonal influences on diving behaviour have been made in some other harbour seal populations, such as in Alaska where seals also dived deeper in the winter compared to summer. In this case the deeper diving was associated with prey availability [86], but physiological and behavioural constraints might also play roles in seasonal patterns [87,88,89]. Winter and spring diets of harbour seals in Svalbard are currently not known, however, the available information from fish surveys conducted in the winter in the Barents Sea and Svalbard waters show that young Atlantic cod (age group 1–3) are present in the WSC through the winter [82,83]. Polar cod are resident in arctic waters year-round. The vertical diel migration pattern disappears during the Polar Night for both of these fish species [83,85]. Concomitantly, the seals diel diving pattern also disappears during the Polar Night suggesting that they adjust to their prey’s change in behaviour [83,85]. Surprisingly, the seals did not resume a diel diving pattern when the light reappeared in March (Fig 3). This suggests that the seals might utilize another prey type or another gadoid age class that does not exhibit diel vertical migration during the spring. Unfortunately, recent attempts to sample diet using scats analysis at this time of year failed, because the haul-out sites were heavily iced and the seals remained in the water or on drift ice. The strong seasonal changes observed in foraging effort (Fig 5) are likely linked to the prey distribution, type or aggregation. Direct observations of prey capture events with cameras have shown that harbour seal prey handling time varies according to the size and species being taken; with large fish being more likely to be consumed at the surface than small prey which can be swallowed hole at depth [78]. Consequently, the time spent at the bottom of dives might be shorter for large prey compared to small schooling fish which are consumed at depth.

Dives also changed throughout the year with respect to bathymetry; less diving took place in shallow coastal areas (<50 m) in the winter while pelagic diving in deeper areas increased and in fact became predominant (Fig 4). These dives over deep water were likely influenced by larger oceanographic processes instead of more local currents occurring in coastal areas. Fish species’ distributions are tightly linked to water temperature and salinity [80]; thus many fish species are limited to certain water types and are therefore influenced by these same oceanographic processes [81,90]. The distribution of fish species around Svalbard is a complex patchwork because the climate and oceanographic conditions surrounding the archipelago change on a relatively fine scale, particularly on the west coast [68]. Frontal regions, such as the PF that occurs on the west coast of Spitsbergen, are generally very productive areas that concentrate phytoplankton, zooplankton and in turn their predators [2,14,91,92]. This seems to be the case along the west coast of Spitsbergen, at least for part of the year where harbour seals concentrated their activity just West of PKF at the PF located about 50 km offshore. Some harbour seals were resident in the area immediately west of PF all year round while others dispersed within the WSS following moulting (Fig 1b).

Concentration of nutrients and plankton also occur within the water column in areas where two water masses are layered and top predators are known to exploit the resulting inter-layer aggregations of prey [93]. Harbour seals in Svalbard seemed to follow this foraging strategy for at least part of the year. They dove down to, or just below, the MLD through the autumn and early winter. But, from set points in the winter or spring (April 2010 and December 2011) in the two study years, respectively, the seals began to dive deeper than the MLD targeting a different water mass (Fig 8). During these periods the recorded dive parameters were at their extremes in terms of duration and depth. This type of diving occurred intermittently, and was likely linked to transient oceanographic features.

The WSS is a particularly dynamic region that is dominated by Atlantic-modified water in the summer/early autumn and by ArW in the winter [47]. This cycle is clearly seen in the CTD data collected by the seals (Figs 6 and 7, S1 Fig). Surface waters become colder due to surface heat loss to the atmosphere when winter approaches and get warmer when air temperature increases again in the spring. This cycle is also apparent to some extent at greater depths where water temperature slowly decreases. However, throughout the winter sharp increases in temperature and salinity occur at irregular intervals at depths of 100–150 m (Figs 6 and 7) via influxes of AW (which is the sole source of heat at depth in this region [82]). AW is typically restricted to ~ 500 m depth in the WSC with strong density gradients at the PF acting as a barrier that generally prevents exchanges between the AW and ArW masses [46]. However, under certain conditions AW can penetrate onto the shelf through upwelling, carrying with it AW-associated fish species [68,83]. Two mechanisms are thought to drive upwelling of AW onto the shelf, wind-driven surface offshore Ekman transport and to a lesser extent instability of barotropic or baroclinic gradients along the front leading to the formation of localised eddies [68,83,94]. These cross-shelf exchanges make this region of Svalbard very dynamic and productive through mixing phenomenon between nutrient-rich AW and local Arctic-like waters [46,81]. Wind-driven upwelling events have been found to be associated with protracted strong northerly or easterly winds occurring mainly in the winter [45,68]. In the present study, northerly or easterly winds occurring the preceding week were found to be correlated with dive parameters of the harbour seals, essentially showing that the animals reacted to upwelling events by altering their diving behaviour. When the WSS was dominated by ArW masses or modified AW masses, the seals mainly targeted the MLD, but when influxes of AW water flooded the WSS, they preferentially targeted this water mass. The seals were likely preying on young Atlantic cod within the AW, brought into their foraging depth range through the upwelling events on the WSS. This would also explain why harbour seals in Svalbard seldom enter deep fjords which are under normal conditions dominated by ArW, and why they seem to avoid the east coast of the archipelago even during periods when it is ice free.

Ice concentration was not retained in any of the top-ranked models describing environmental impacts on the diving behaviour of the seals. However, drift ice is most certainly affected by winds and it has been suggested that influxes of warm AW might impact sea ice formation and enhance melting processes when the heat contained in the water column is released at the surface [68,94]. Additionally, changes in ice concentration and upwelling do not necessarily operate on the same time scale; drift ice in light to moderate concentrations reacts almost immediately to wind forcing, while sustained winds are needed to trigger upwelling [68]. This likely explains why the seals’ diving behaviour was not directly influenced by sea ice concentrations independently, although they do actively avoid areas with very high ice concentrations [34].

Climate change has already had a major impact on air and surface temperatures and sea ice thickness and extent in Svalbard [32,83,95]. The warming of the Svalbard region is predicted to continue and even intensify in the coming years and changes such as higher sea surface temperatures, increased frequency of strong winds and increased temperatures of the core AW are expected [46,83]. Less ice on the west coast of Spitsbergen will further enhance the effect of wind-induced mixing of the water column and will cause stronger upwelling events [96]. Such events have already been observed; in 2012 during a winter cruise at the ice edge North of Spitsbergen an open water area that would have normally have been ice-covered, was flooded by AW [46]. Increased mass transport of AW by the WSC will lead to an increase in the presence of temperate species on the west coast of Svalbard and induce major changes in the food web [81,82]. Typical Atlantic species such as Atlantic cod, haddock, Atlantic salmon *(Salmo salar)*, capelin *(Mallotus villosus)* and mackerel have already been observed in the fjords on the west coast of Spitsbergen [82,97]. These temperate species are now co-occurring on the WSS with typical arctic species such as polar cod leading to potential new ecological interactions.

## Conclusions

This study presents novel telemetry data from harbour seals spanning from post-moulting to the next breeding season in combination with small-scale hydrographic data collected by the seals themselves allowing for exploration of fine-scale movements in comparison to environmental conditions. Behavioural data from the CTD-SRDLs instrumented seals showed that their diving patterns were affected by seasonally changing environmental parameters. Dives were deeper, longer and less numerous, with a proportionally shorter bottom time under conditions created by wind-driven upwelling events in winter. During these events the WSS was flooded with warm saline AW bringing AW-associated prey species into reach of the harbour seals. This seal species is essentially a temperate species that is not normally found in High Arctic regions. But, the presence of AW and the lack of land-fast ice, combined with the richness and productivity of the WSS arising from the intense mixing of water masses in this region, are factors that make it possible for a population of harbour seals to be resident at this high latitude. The local oceanographic features and processes also explain why this population is limited to the west coast of Spitsbergen within the Svalbard Archipelago. Increased influxes of AW and decreased sea-ice cover that are predicted to occur in the future are likely to enhance the mixing in the water column and increase the abundance of Atlantic fish and zooplankton species, which in turn is likely to favour growth of this harbour seal population.

## Supporting Information

S1 Fig Temporal changes in temperature at different depths across the study region from September through May for both study years. The monthly temperature profiles were collected by 30 adult and juvenile harbour seals equipped with Conductivity-Temperature-Depth Satellite-Relay-Data-Loggers (CTD-SRDLs) during 2009/2010 and 2010/2011 in Svalbard, Norway. The temperature isosurfaces at 6 m, 50 m, 100 m and 150 m are presented.
(ZIP)

S1 Table Summary of morphometric information. Morphometric information, and record duration, for 30 adult and juvenile harbour seals equipped with Conductivity-Temperature-Depth Satellite-Relay-Data-Loggers (CTD-SRDLs) in Svalbard, Norway during 2009/2010 and 2010/2011. *Ad* Adult, *Juv* Juvenile, *M* Male, *F* Female.
(DOCX)

S2 Table Model selection table describing the probability of a dive belonging to a bathymetry category. The Bayesian Information Criterion (BIC), change in BIC and deviance are presented for the top multinomial models exploring the relationship between the probability for a dive to belong to a bathymetry category for 30 adult and juvenile harbour seals equipped with Conductivity-Temperature-Depth Satellite-Relay-Data-Loggers (CTD-SRDLs) in Svalbard, Norway during 2009/2010 and 2010/2011. The null model is also presented for comparative purposes.
(DOCX)
