# Supplementary figures and images for: Seasonal, Oceanographic and Atmospheric Drivers of Diving Behaviour in a Temperate Seal Species Living in the High Arctic
Source: PLoS One. 2015 Jul 21;10(7):e0132686. doi: 10.1371/journal.pone.0132686 (PMC4509669; doi:10.1371/journal.pone.0132686)

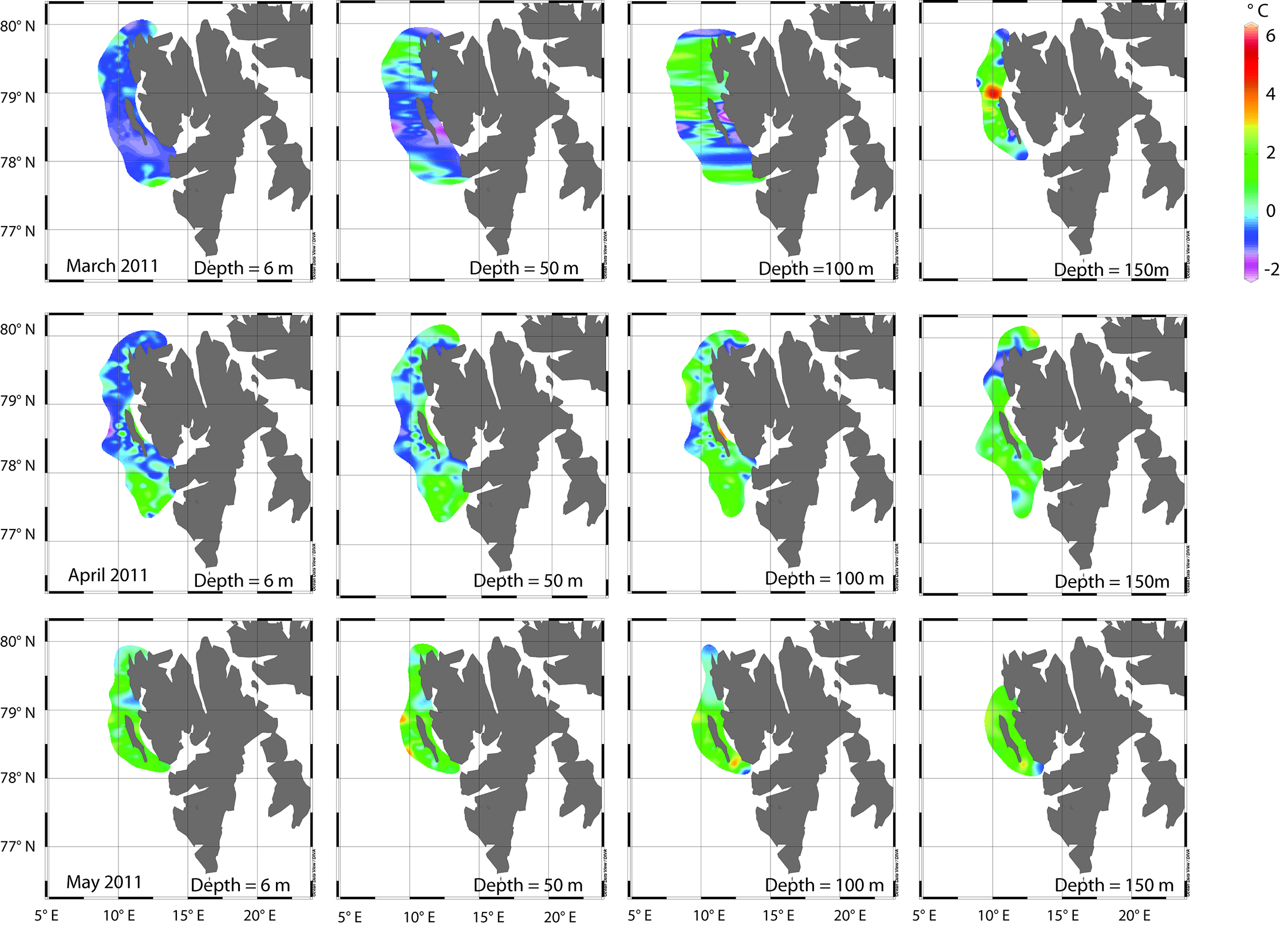

Supplement: S1 Fig — The monthly temperature profiles were collected by 30 adult and juvenile harbour seals equipped with Conductivity-Temperature-Depth Satellite-Relay-Data-Loggers (CTD-SRDLs) during 2009/2010 and 2010/2011 in Svalbard, Norway. The temperature isosurfaces at 6 m, 50 m, 100 m and 150 m are presented. (ZIP) [file pone.0132686.s001.zip › S1f_Figure_publication.tif]

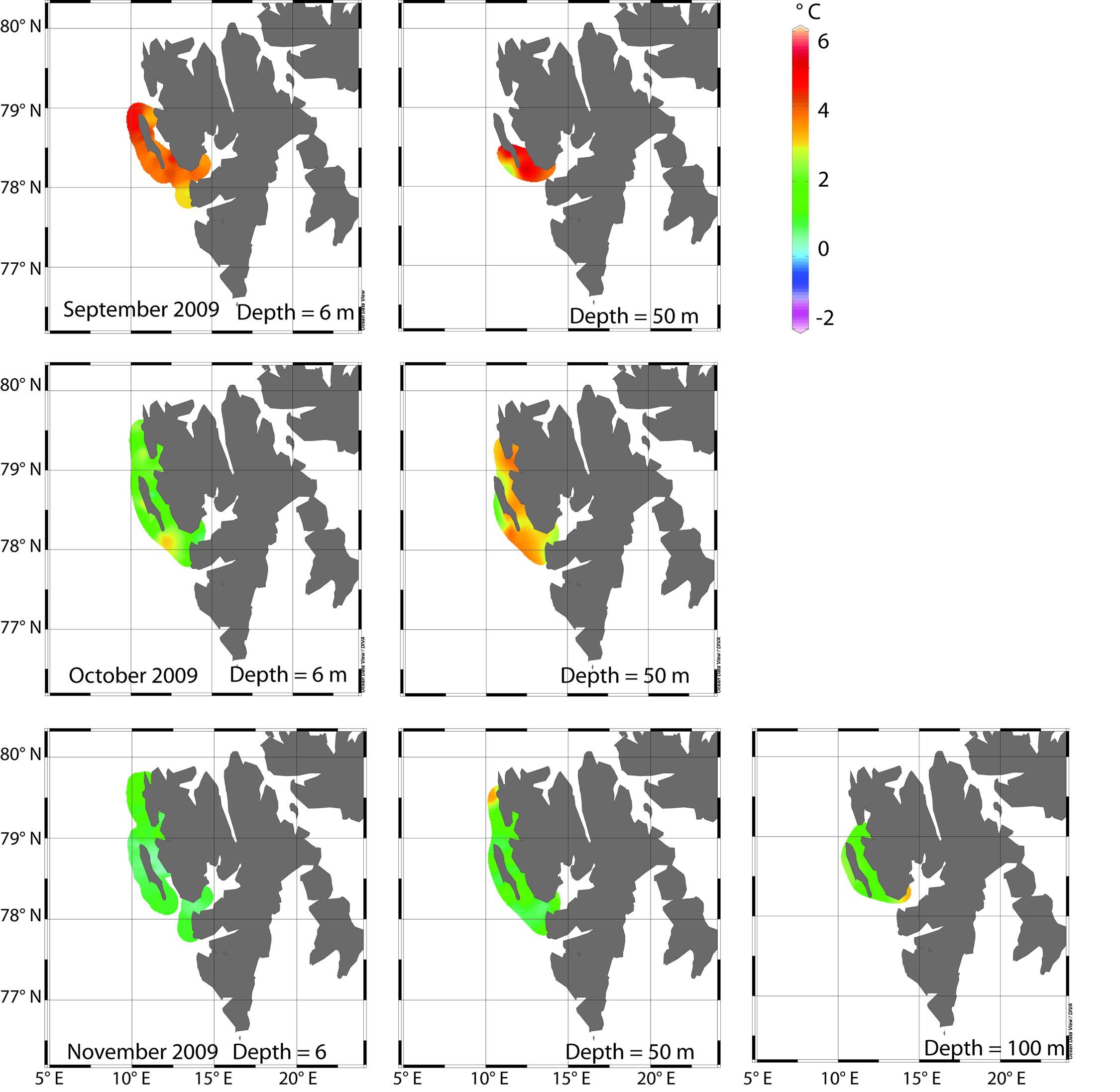

Supplement: S1 Fig — The monthly temperature profiles were collected by 30 adult and juvenile harbour seals equipped with Conductivity-Temperature-Depth Satellite-Relay-Data-Loggers (CTD-SRDLs) during 2009/2010 and 2010/2011 in Svalbard, Norway. The temperature isosurfaces at 6 m, 50 m, 100 m and 150 m are presented. (ZIP) [file pone.0132686.s001.zip › S1a_Figure_publication.tif]

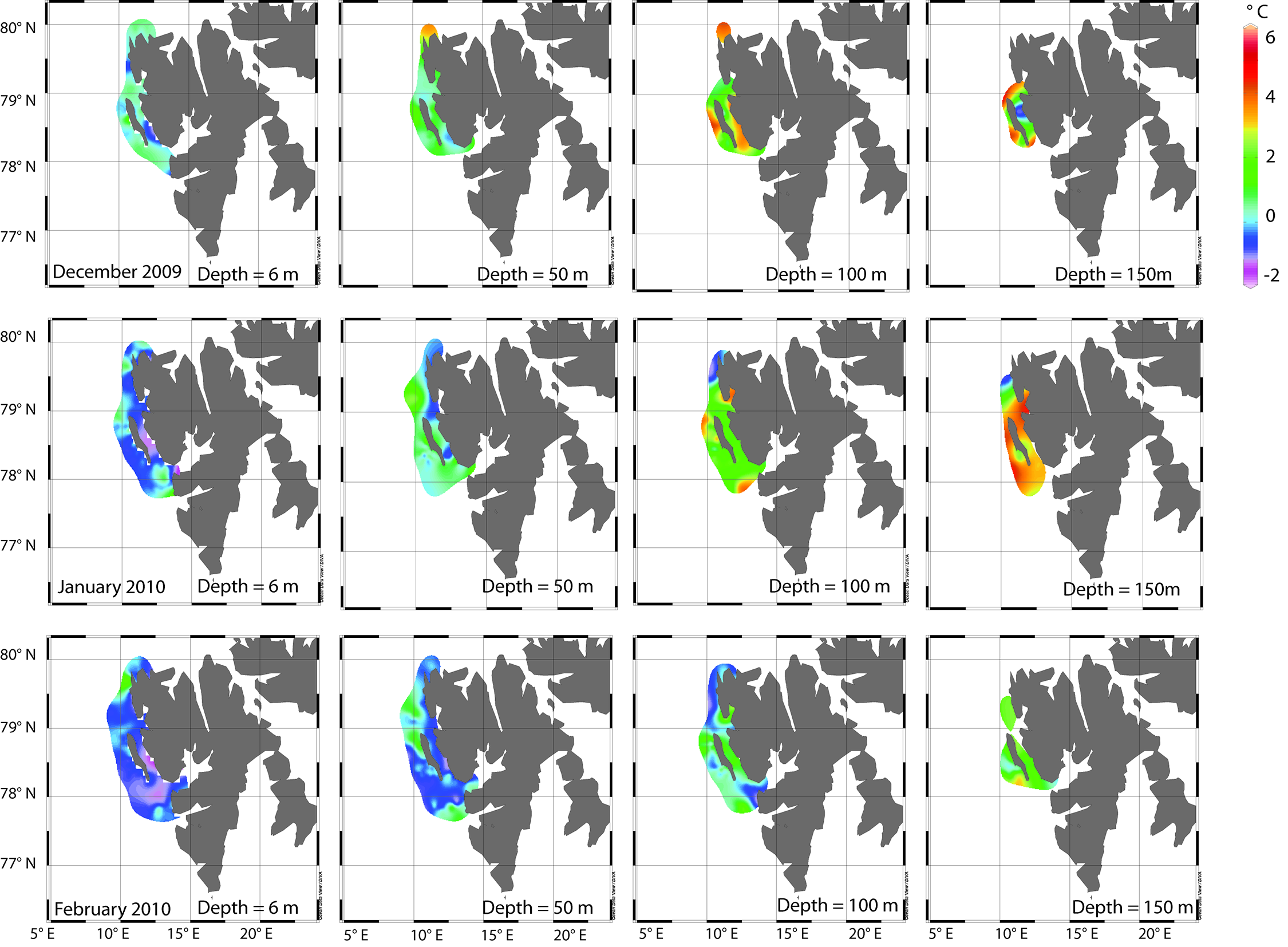

Supplement: S1 Fig — The monthly temperature profiles were collected by 30 adult and juvenile harbour seals equipped with Conductivity-Temperature-Depth Satellite-Relay-Data-Loggers (CTD-SRDLs) during 2009/2010 and 2010/2011 in Svalbard, Norway. The temperature isosurfaces at 6 m, 50 m, 100 m and 150 m are presented. (ZIP) [file pone.0132686.s001.zip › S1b_Figure_publication.tif]

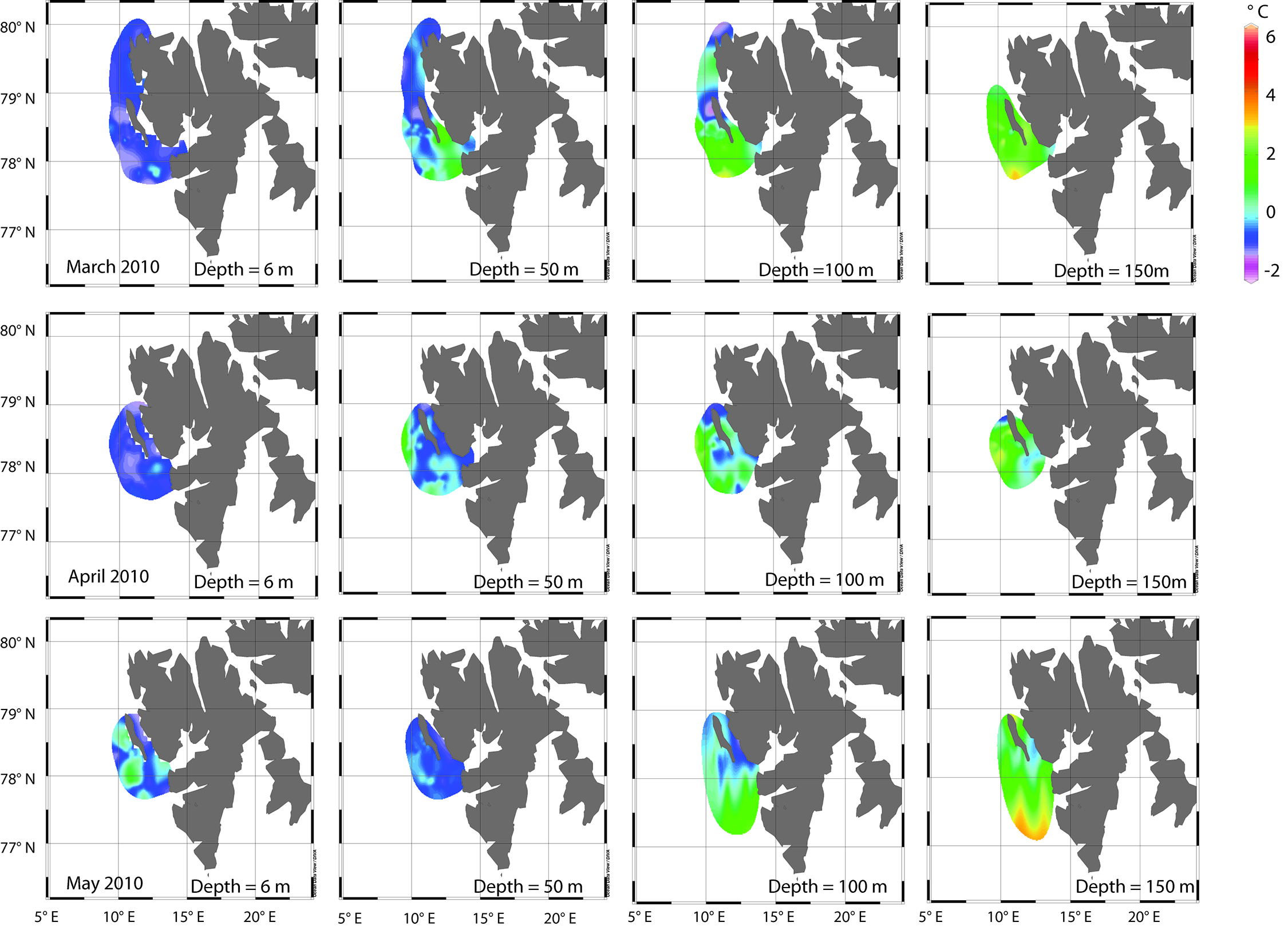

Supplement: S1 Fig — The monthly temperature profiles were collected by 30 adult and juvenile harbour seals equipped with Conductivity-Temperature-Depth Satellite-Relay-Data-Loggers (CTD-SRDLs) during 2009/2010 and 2010/2011 in Svalbard, Norway. The temperature isosurfaces at 6 m, 50 m, 100 m and 150 m are presented. (ZIP) [file pone.0132686.s001.zip › S1c_Figure_publication.tif]

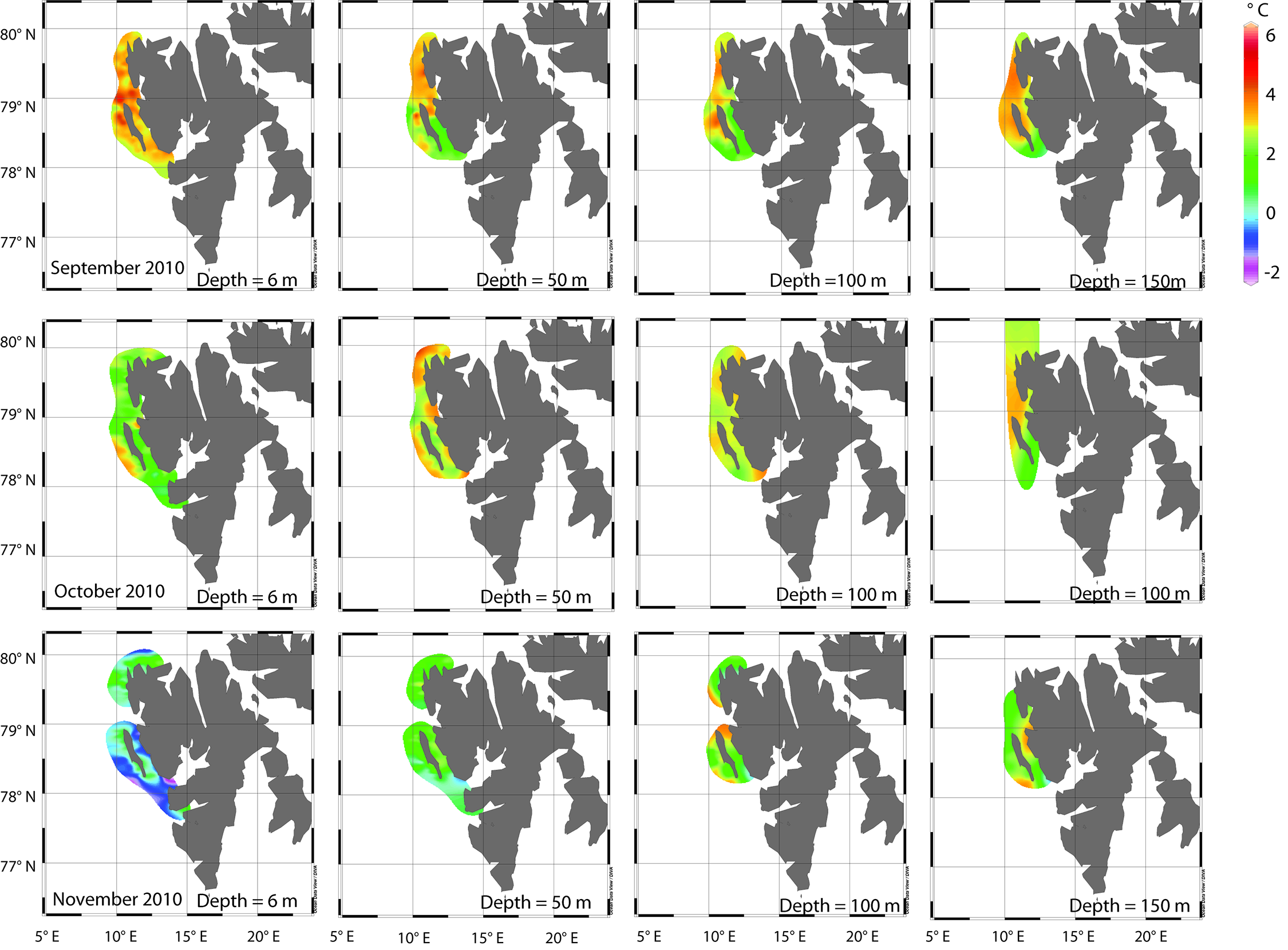

Supplement: S1 Fig — The monthly temperature profiles were collected by 30 adult and juvenile harbour seals equipped with Conductivity-Temperature-Depth Satellite-Relay-Data-Loggers (CTD-SRDLs) during 2009/2010 and 2010/2011 in Svalbard, Norway. The temperature isosurfaces at 6 m, 50 m, 100 m and 150 m are presented. (ZIP) [file pone.0132686.s001.zip › S1d_Figure_publication.tif]

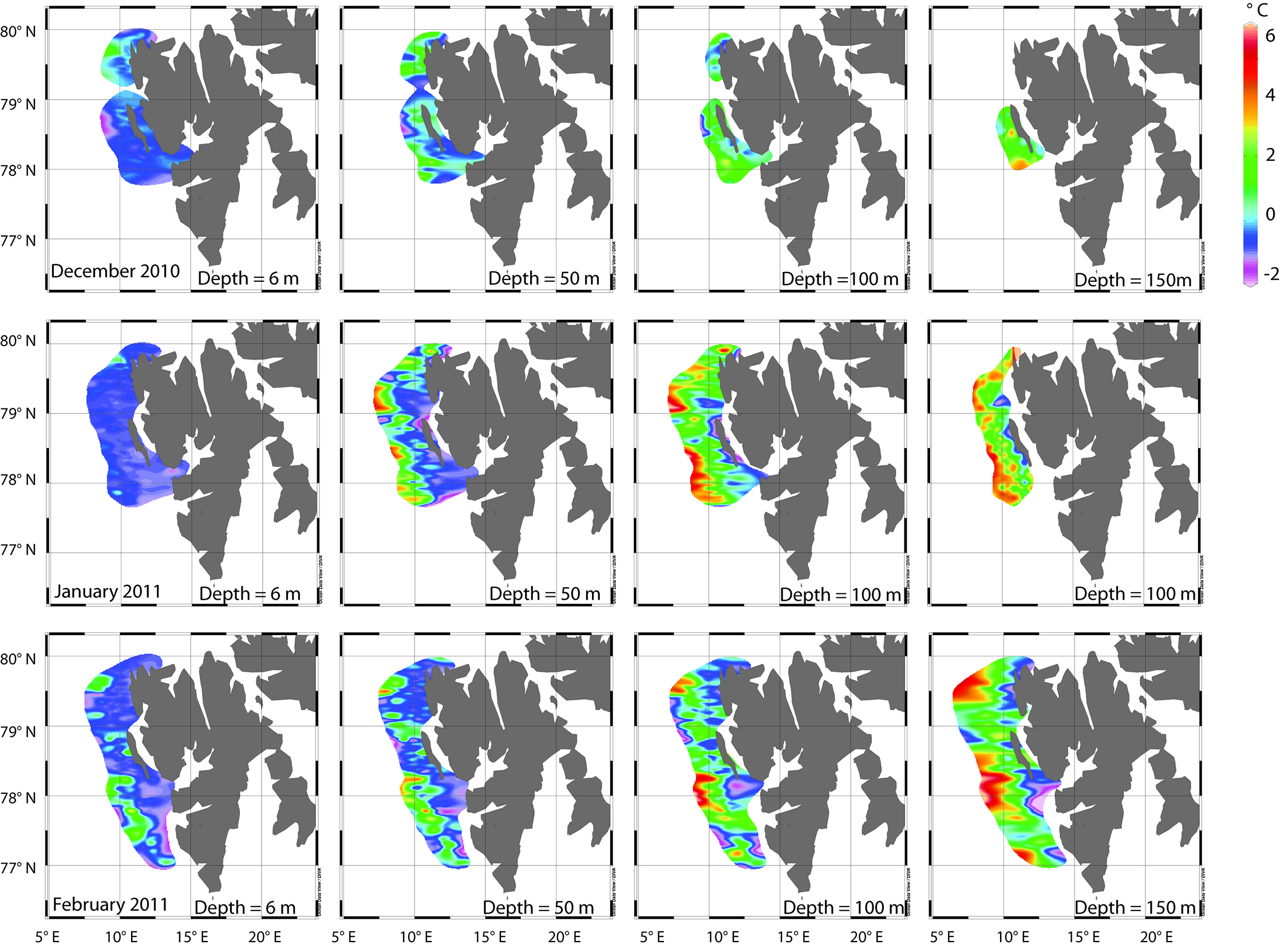

Supplement: S1 Fig — The monthly temperature profiles were collected by 30 adult and juvenile harbour seals equipped with Conductivity-Temperature-Depth Satellite-Relay-Data-Loggers (CTD-SRDLs) during 2009/2010 and 2010/2011 in Svalbard, Norway. The temperature isosurfaces at 6 m, 50 m, 100 m and 150 m are presented. (ZIP) [file pone.0132686.s001.zip › S1e_Figure_publication.tif]
