# Supplementary material for: Seasonal, Oceanographic and Atmospheric Drivers of Diving Behaviour in a Temperate Seal Species Living in the High Arctic
Source: PLoS One. 2015 Jul 21;10(7):e0132686. doi: 10.1371/journal.pone.0132686 (PMC4509669; doi:10.1371/journal.pone.0132686)
Supplement: S1 Table — Morphometric information, and record duration, for 30 adult and juvenile harbour seals equipped with Conductivity-Temperature-Depth Satellite-Relay-Data-Loggers (CTD-SRDLs) in Svalbard, Norway during 2009/2010 and 2010/2011. Ad Adult, Juv Juvenile, M Male, F Female. (DOCX) [file pone.0132686.s002.docx]

**Table S1. Summary of morphometric information.** Morphometric information, and record duration, for 30 adult and juvenile harbour seals equipped with Conductivity-Temperature-Depth Satellite-Relay-Data-Loggers (CTD-SRDLs) in Svalbard, Norway during 2009/2010 and 2010/2011

| **Seal ID** | **Deployment Date** | **Age** | **Group** | **Sex** | **Weight** | **Girth** | **Length** | **Record duration (days)** |
| --- | --- | --- | --- | --- | --- | --- | --- | --- |
| F41 | 02/09/2009 | 2 | Juv | F | 41 | 90 | 104 | 278 |
| F44 | 02/09/2009 | 2 | Juv | F | 44 | 89 | 116 | 146 |
| F47 | 02/09/2009 | 2 | Juv | F | 47 | 92 | 118 | 128 |
| F48 | 01/09/2009 | 3 | Juv | F | 48 | 93 | 120 | 100 |
| F60 | 02/09/2009 | 4 | Ad | F | 60 | 98 | 126 | 200 |
| F66 | 03/09/2009 | 5 | Ad | F | 66 | 100 | 133 | 96 |
| F74 | 01/09/2009 | 11 | Ad | F | 74 | 108 | 139 | 159 |
| F76 | 04/09/2009 | 9 | Ad | F | 76 | 107 | 139 | 279 |
| M43 | 04/09/2009 | 2 | Juv | M | 43 | 86 | 113 | 176 |
| M51 | 04/09/2009 | 2 | Juv | M | 51 | 96 | 120 | 278 |
| M52 | 05/09/2009 | 2 | Juv | M | 52 | 98 | 122 | 276 |
| M56 | 04/09/2009 | 4 | Ad | M | 56 | 101 | 128 | 282 |
| M64 | 06/09/2009 | 3 | Ad | M | 64 | 94 | 130 | 255 |
| M65 | 14/09/2009 | 3 | Ad | M | 65 | 109 | 128 | 267 |
| M77 | 10/09/2009 | 5 | Ad | M | 77 | 111 | 142 | 106 |
| F42 | 26/08/2010 | 2 | Juv | F | 42 | 88 | 109 | 148 |
| F44 | 28/08/2010 | 3 | Juv | F | 44 | 88 | 116 | 143 |
| F50 | 23/08/2010 | 3 | Juv | F | 50 | 87 | 127 | 54 |
| F53 | 03/09/2010 | 4 | Ad | F | 53 | 92 | 120 | 156 |
| F58a | 25/08/2010 | 5 | Ad | F | 58 | 95 | 124 | 85 |
| F58b | 28/08/2010 | 5 | Ad | F | 58 | 94 | 132 | 296 |
| F59 | 04/09/2010 | 3 | Juv | F | 59 | 93 | 125 | 102 |
| M41 | 25/08/2010 | 1 | Juv | M | 41 | 89 | 105 | 284 |
| M45 | 31/08/2010 | 2 | Juv | M | 45 | 86 | 122 | 150 |
| M48 | 25/08/2010 | 3 | Juv | M | 48 | 86 | 128 | 286 |
| M53a | 31/08/2010 | 3 | Juv | M | 53 | 97 | 126 | 291 |
| M53b | 03/09/2010 | 2 | Juv | M | 53 | 93 | 123 | 175 |
| M57 | 24/08/2010 | 5 | Ad | M | 57 | 96 | 132 | 298 |
| M64 | 26/08/2010 | 4 | Ad | M | 64 | 104 | 127 | 288 |
| M65 | 24/08/2010 | 5 | Ad | M | 65 | 95 | 137 | 234 |

^*^*Ad* Adult, *Juv* Juvenile, *M* Male, *F* Female
