# Supplementary material for: Seasonal, Oceanographic and Atmospheric Drivers of Diving Behaviour in a Temperate Seal Species Living in the High Arctic
Source: PLoS One. 2015 Jul 21;10(7):e0132686. doi: 10.1371/journal.pone.0132686 (PMC4509669; doi:10.1371/journal.pone.0132686)
Supplement: S2 Table — The Bayesian Information Criterion (BIC), change in BIC and deviance are presented for the top multinomial models exploring the relationship between the probability for a dive to belong to a bathymetry category for 30 adult and juvenile harbour seals equipped with Conductivity-Temperature-Depth Satellite-Relay-Data-Loggers (CTD-SRDLs) in Svalbard, Norway during 2009/2010 and 2010/2011. The null model is also presented for comparative purposes. (DOCX) [file pone.0132686.s003.docx]

**Table S2. Model selection table describing the probability of a dive belonging to a bathymetry category**. The Baysian Information Criterion (BIC), change in BIC and deviance are presented for the top multinomial models exploring the relationship between the probability for a dive to belong to a bathymetry category for 30 adult and juvenile harbour seals equipped with Conductivity-Temperature-Depth Satellite-Relay-Data-Loggers (CTD-SRDLs) in Svalbard, Norway during 2009/2010 and 2010/2011. The null model is also presented for comparative purposes.

| **Model structure** | **BIC** | **Δ BIC** | **Deviance** |
| --- | --- | --- | --- |
| ***month*year*** | 241093.4 | 0.00 | 254343.6 |
| *month+age+sex+year* | 245819 | 4725.60 | 245503.1 |
| *month+age+year* | 249058.4 | 7965.00 | 248766.8 |
| *month+year* | 251365.5 | 10272.10 | 251098.2 |
| *month* | 254586.6 | 13493.20 | 240607.5 |
| *~1* | 312872 | 71778.60 | 312848.1 |
